# Multi-faceted examination of a deepwater seamount reveals ecological patterns among coral and sponge communities in the equatorial Pacific

**DOI:** 10.1038/s41598-025-86163-z

**Published:** 2025-01-17

**Authors:** Brian RC Kennedy, Steven Auscavitch, Timothy M. Shank, Constance Sartor, Anameere Tennaba, Alexis M. Weinnig, Randi D. Rotjan

**Affiliations:** 1 Department of Biology, Boston University, Boston, MA USA; 2 Biology Department, Woods Hole Ocean Oceanographic Institution, Woods Hole, MA USA; 3 University of Guam, Mangilao, GU USA; 4 Eastern Ecological Science Center, United States Geological Survey, Kearnesville, WV USA; 5 GeoScience Division ECOP Officer, Ministry of Fisheries and Marine Resources Development, Republic of Kiribati, Tarawa, Kiribati; 6 Blue Nature Alliance, Arlington, VA, USA

**Keywords:** Seamount, Pacific Islands Heritage, Deep-sea coral, Vertical zonation, Deep-sea sponges, Community composition, Vulnerable marine ecosystems, Biodiversity, Community ecology

## Abstract

Spatial changes in benthic community structure have been observed across natural gradients in deep-sea ecosystems, but these patterns remain under-sampled on seamounts. Here, we identify the spatial composition and distribution of coral and sponge taxa on four sides of a single central Pacific equatorial “model” seamount within the US EEZ surrounding the Howland and Baker unit of the Pacific Islands Heritage Marine National Monument. This seamount rises from 5,000 + m to mesophotic depths of 196 m, and is influenced by the Equatorial Undercurrent. Four remotely operated vehicle (ROV) transects were completed, one on each flank of the seamount. Shallower than ~ 250 m, the mesophotic seafloor was composed of scoured carbonate pavement with sediment accumulation only found in rocky depressions. Waters below 500 m hosted communities predominantly composed of octocorals, however, several coral taxa showed seamount flank preference (higher abundance on one or more flanks than the others) even though strong vertical (depth) zonation of corals and sponges was observed on all flanks. Euplectellidae, Plexauridae and ​​*Chrysogorgia* spp. corals each showed a distinct preference for flank. To help visualize the influence of current flow, oxygen, depth, and substrate type on the zonation of seamounts, we created an Alexander Von Humboldt-style infographic to illustrate the observed biodiversity patterns. Given the importance of seamounts to ocean biodiversity and productivity, this study is an early attempt at a holistic visualization of seamount biology that can advance new hypotheses about seamount ecology.

**Supplementary Information:**

The online version contains supplementary material available at 10.1038/s41598-025-86163-z.

## Introduction

Resolving patterns of biological community structure on individual seamounts is of continued interest and increasing ecological importance^1^, as more deep-sea communities are under increasing threat from anthropogenic factors^2^. The increased number of ships and other deep submergence assets have resulted in a notable increase in visual seafloor survey data that now make it possible to investigate deep-sea biological communities at a previously unprecedented scale. To-date, it has been shown that community distributions reveal biogeographic patterns on the scale of individual seamounts, demonstrating that depth is a major driver of benthic distribution at the species, genus, and family level^3,4^. Further, correlations between depth and other oceanographic conditions have also been investigated, and it is clear that depth is not the only abiotic factor of importance^5–8^. While individual seamounts may be biologically distinct^9,10^, and foster varying degrees of endemism^11^, recent bursts of exploratory expeditions have shown a wider geographic spread of many taxa within a region, thereby suggesting that there may be ecological organization of biological communities on seamounts that may share similarities across a regional scale^12^. For example, Kennedy and Rotjan^13^showed that some benthic coral taxa in the Pacific have an affinity for specific seamount shapes. Similarly, Summers and Watling^14^ found that there are distinct biogeographic provinces that demonstrate ranges of specific benthic taxa. Yet, these patterns have mostly been derived from single transect lines on a single seamount, and there have been limited attempts to characterize benthic communities across a seamount from multiple flanks.

To our knowledge, no seamount has been comprehensively assessed (every square meter), and few studies have investigated a single seamount holistically, examining faunal distribution around the seamount via multiple transects on different sides. One example of relatively holistic analysis comes from Morgan, et al.^15^, who described the assemblages on flanks of Mokumanamana Island in Hawaii and found that different sides of the feature hosted different species assemblages. Conversely, Du Preez et al.^16^ reported more similarity across the different sides of Cobb seamount, a North Pacific seamount, but with depth being a greater influence than seamount side (flank). However, both studies documented heterogeneity in the communities across flanks, and both studies assigned communities to assemblages and proposed biological zones.

These putative biological zones may be viewed as early tools to establish hypotheses of seamount community organization that can serve to test ecological drivers of benthic community patterns. However, so few seamounts have been investigated on multiple flanks that examining consistent patterns of zonation is not yet possible at or beyond the regional scale. Essentially, we are at the same point in our understanding of seamount ecology as when Alexander von Humboldt, Alexander Keith Johnston, and Aimé Bonpland first boldly articulated hypothesized zones of plant life on terrestrial mountains in the early 1800s. This collaborative team together created some of the first scientific infographics of how ecosystems and communities changed as a function of elevation and wind direction on volcanic mountains in the Andes^17^. Those early infographics provided a visually compelling and artful framework that propelled the biological community forward - daring the community to provide data to either support or refute their understanding of botanical organization on a mountain-by-mountain scale, which then led to the genesis of zone differentiation by elevation and other abiotic contributors. However, 200 years later, a similar effort has not yet been undertaken for underwater seamounts.

Seamounts are as diverse as mountains, but suffer from a lack of feature-specific data. One 2010 study estimated that less than 1% of seamounts had been imaged and the vast majority had seafloor data from only one imaging transect, along a single flank, covering only a limited depth range^18^. This chronic under-sampling is largely due to the cost of operating deep submergence vehicles and the remoteness of seamounts^18^. Given the challenges of working in the deep sea, a model seamount is needed to answer questions about community changes across a single feature. We define a model seamount as one that would be simple, relatively symmetric without strong, distinctive bathymetric features, and would cover a wide range of depths. Further, we envision a strong model seamount as one with relatively simplistic morphological features that could be contrasted with strong environmental gradients such as strong surface current flow or changes in dissolved oxygen across the range of depths that would help test the hypotheses related to the drivers of community composition.

In this study, we tested whether or not spatial benthic biodiversity patterns were the same regardless of flank and depth on a relatively small, comparatively smoothly shaped seamount that fits the characteristics of a “model” seamount (described above) in the equatorial Pacific in U.S. waters in the area surrounding the Howland and Baker unit of the Pacific Islands Heritage^19^ (formerly Pacific Remote Islands) Marine National Monument (1.610 N, 175.203 W). This seamount is located in the equatorial undercurrent, which has strong surface currents running predominantly west-to-east. We investigated the local contribution of beta diversity for depth bins for deep-water corals and sponges. To assess the influence of abiotic factors we measured physical oceanographic parameters across the depth range (temperature, salinity, and dissolved oxygen) as well as current flow from the surface to 600 m depth. We also estimated seafloor slope angles to determine its influence on benthic animal habitat preference. Using video data from four deep-water ROV dives, we described the vertical transitions between communities along transects on different sides and along the top of this conical seamount. We synthesized these data to construct a Von Humboldt-style infographic to illustrate putative bands of benthic communities around the seamount, with the aim of advancing seamount ecology towards a deeper understanding of community organization at the seamount scale.

## Results

### Physico-chemical oceanographic finding

**Fig. 1:**
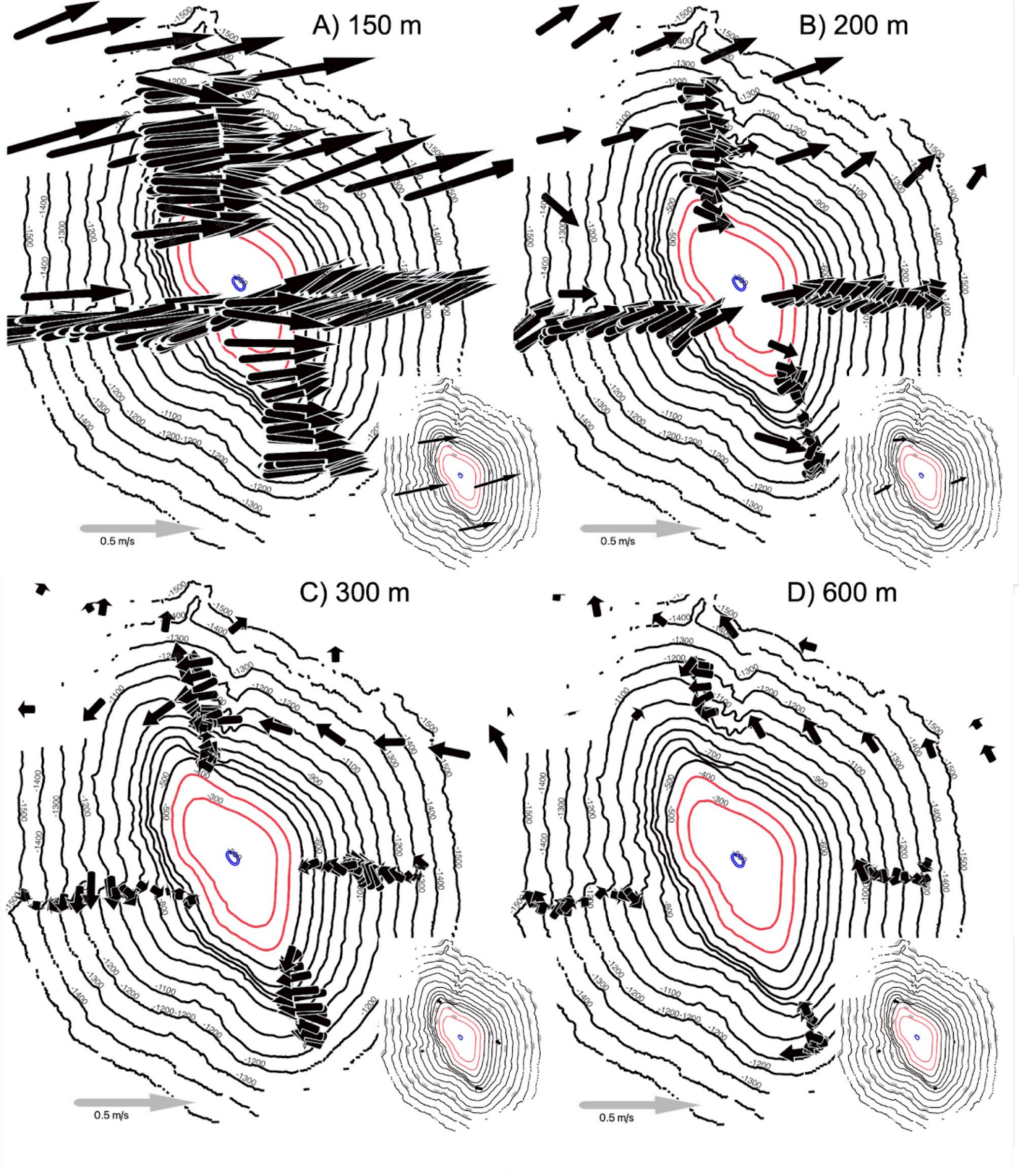
75 kHz Acoustic current doppler current profiler (ADCP) data collected from the hull mounted transducer from the R/V *Falkor*. Arrow direction shows the direction of current flow. The length of the arrow indicated the relative velocity of the water mass at that location. (**A**) Water flows at 150 m, which is above the shallowest point of the seamount. (**B**) 200 m depth, which is the shallowest point of the seamount. (**C**) depth ~ 300 m, there is a strong current reversal at this depth where the water switches from predominantly flowing West to East to East to West. (**D**) Depth 600 m. This is the deepest depth the ADCP data was reliable across most of the seamount and corresponds with the oxygen minimum zone. Note the turbulence in the lee of the seamount on the West side.

The 75 kHz acoustic doppler current profiler data (ADCP) showed several distinct current regimes that changed with depth over the target seamount (Fig. 1). Above the feature, the dominant current flow progressed from West to East in excess of 0.5 m/s, which is consistent with the Equatorial Undercurrent^20^ (Fig. 1A). The summit of the seamount (196 m) is still in the flow of the Equatorial Undercurrent, however the velocity reduced noticeably from the shallower waters above the feature (Fig. 1B). Below 250 m, the current flow switched direction 180 degrees from East to West. By 300 m depth, there was a noticeable difference between the current flow on the North and south seamount flanks compared to the East and West(Fig. 1C). The North and South flanks experienced a moderate flow ~ 0.2 m/s that was consistently East to West while the east and west flanks experienced lower flow and the direction of the flow changed as the distance increased from the sides of the seamount indicating greater turbulence on these two sides of the feature. By 600 m - the maximum effective depth of the ADCP given the conditions during our survey - the overall velocity was greatly reduced and the general flow became South to North, but there were also localized variations depending on distance from the seamount (Fig. 1D).

**Fig. 2:**
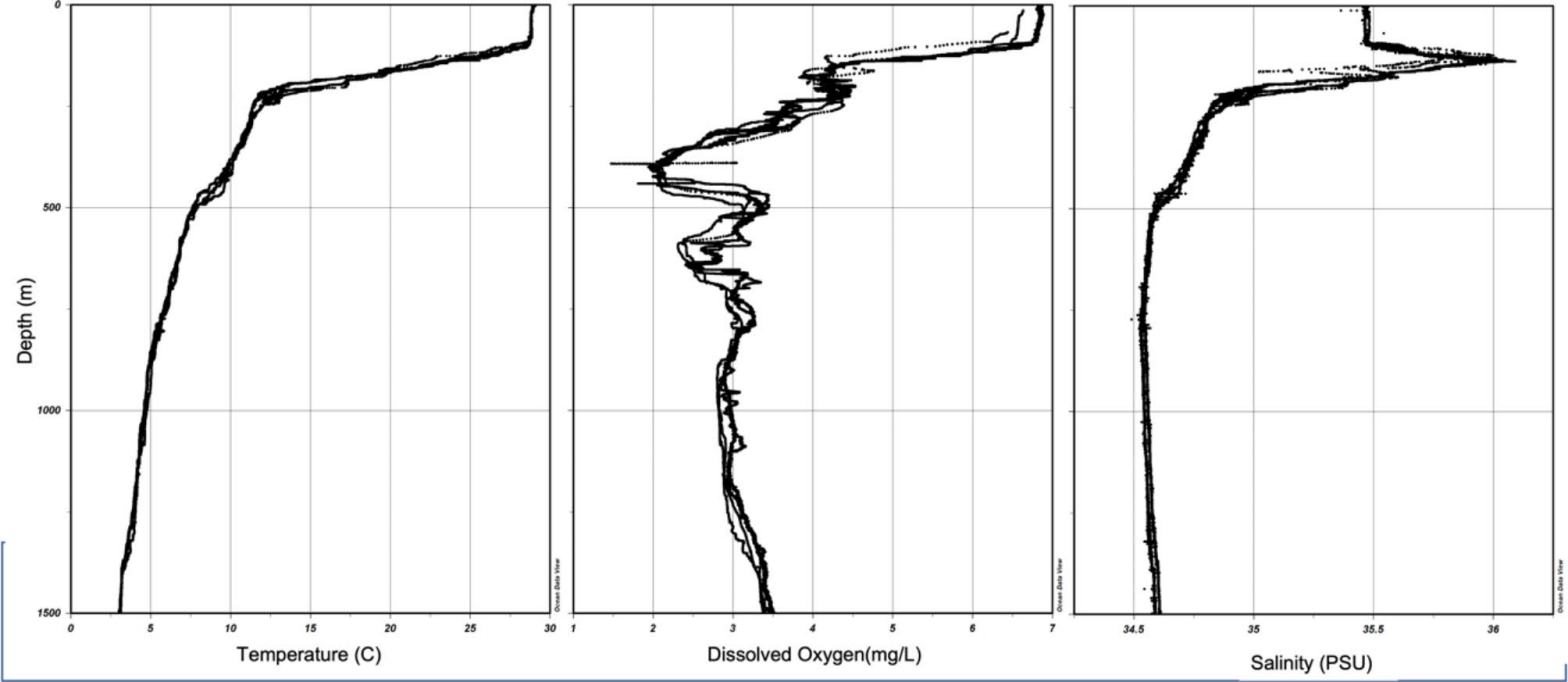
Temperature, oxygen, and salinity profiles. Data is merged from two SBE 49 casts conducted from the ships CTD along with SBE 32 that is mounted on the ROV. ROV CTD data were lost from part of S0433 and all of S0334 thought S0336 due to a mechanical failure, which prompted the two SBE 49 casts to compensate. The left panel is temperature presented in degrees Celsius. The right is the salinity in practical salinity units (PSU). The middle is dissolved oxygen displayed in mg/L units. This data was collected over a five-day window and may not represent longer term average conditions.

**Fig. 3:**
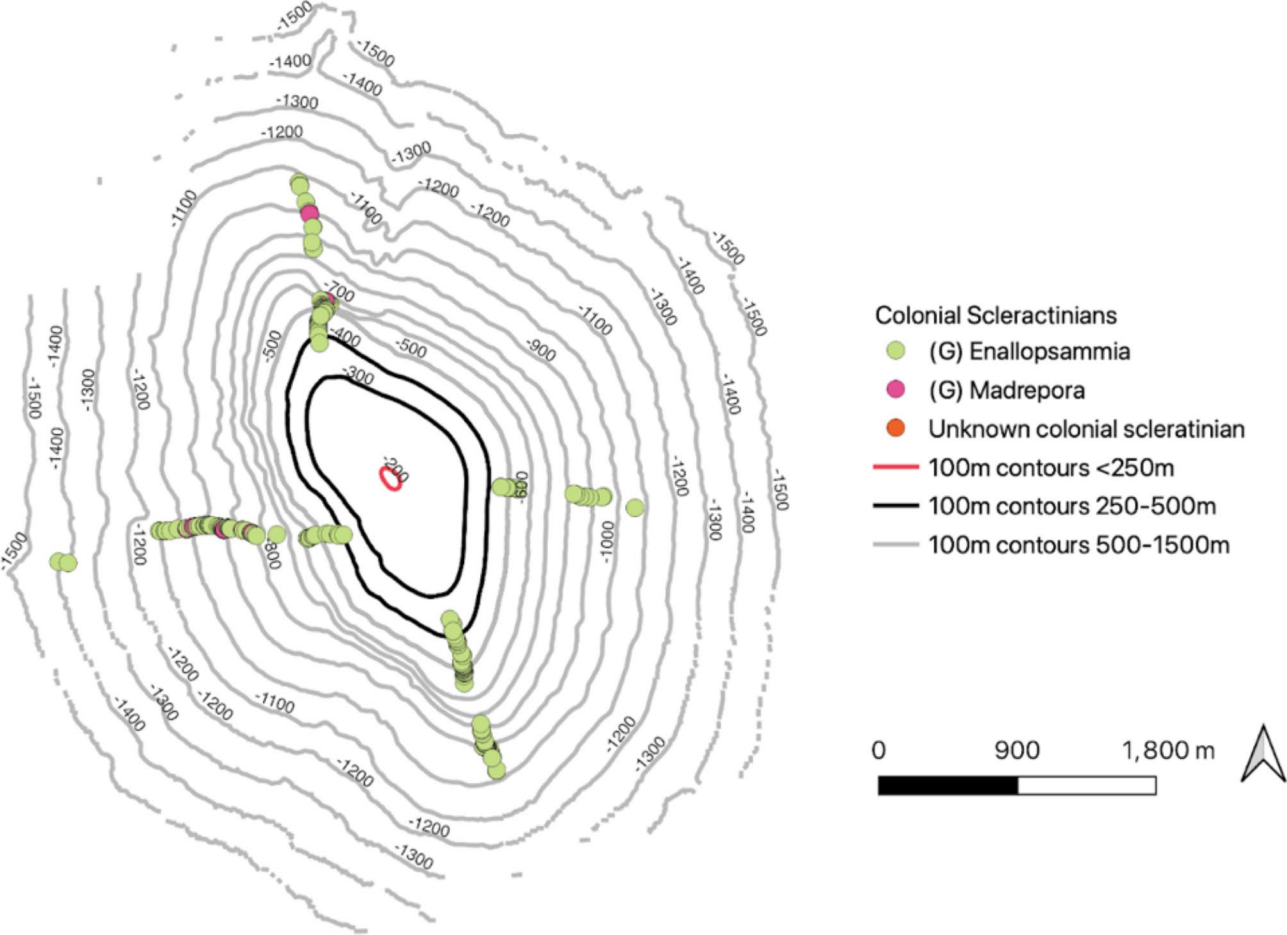
Distribution of colonial Scleractinians on all sides of the feature. There is a gap in their distribution between ~ 500 and ~ 800 m depth which corresponds to the two oxygen minima that can be seen in Fig. 2.

CTD data of the area showed minimal variation around the seamount for temperature and salinity (Fig. 2). However, we noted significant variability in oxygen concentration throughout the water column. We identified two oxygen minima, one at approximately 450 m and a second one at approximately 600 m depth. However, when in contact with the seafloor, these oxygen minima merged to form one larger OMZ between 400 and 700 m on all flanks of the seamount. These oxygen minima seem to have an impact on colonial scleractinians such as *Enallopsammia* and *Madrepora*spp., which have been shown to be oxygen sensitive^21,22^. There was a circumferential band between 500 and 800 m around the seamount where almost no colonial scleractinians were documented (Fig. 3); this gap corresponded with the observed oxygen minimum.

## ROV observations

**Fig. 4:**
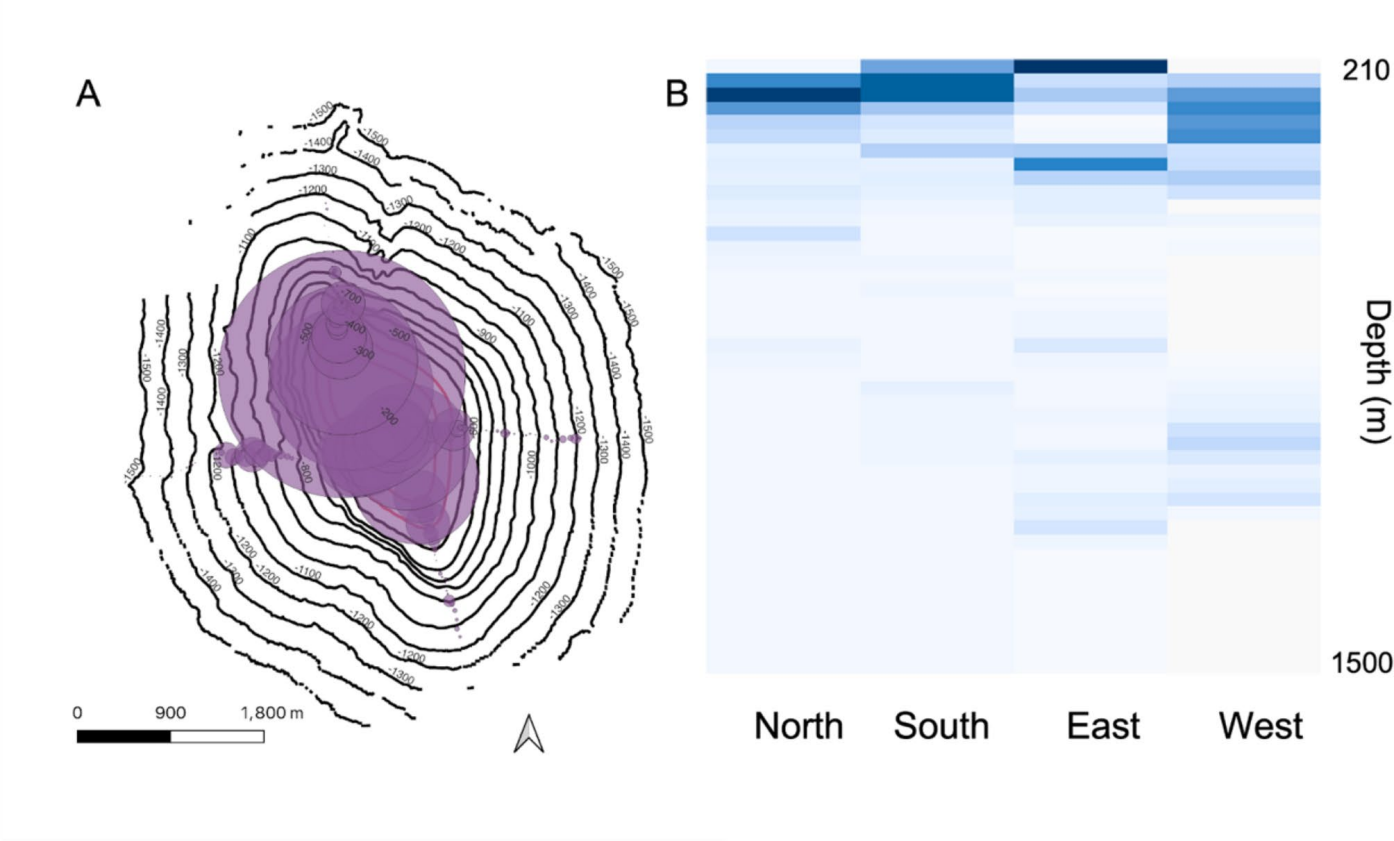
Total abundance of benthic taxa. (**A**) Total number of individuals observed across all taxa based on 30-meter depth bins. (**B**) Heatmaps of total number of individuals based on 30-meter depth bins.

In total, 18,123 individual organisms were identified from 52.5 h of ROV bottom time across the 4 dives. There were differences in community composition and abundance noted on all flanks of the seamount. In general, total abundance was higher near the top of the feature with lower abundance in deeper water (Fig. 4A). The North and West transects showed the greatest abundance across all taxa, but the depths at which the highest abundance was found varied between flanks of the feature. Specifically, the East transect had the lowest abundance and the majority of the organisms documented were found between 200 and 380 m, which contrasts with the western face where the majority of the abundances were found in between the two shallowest depth bands of the western side (Fig. 4B). The North and South transects were more similar to each other with maximum abundance found between 200 and 300 m.

**Fig. 5:**
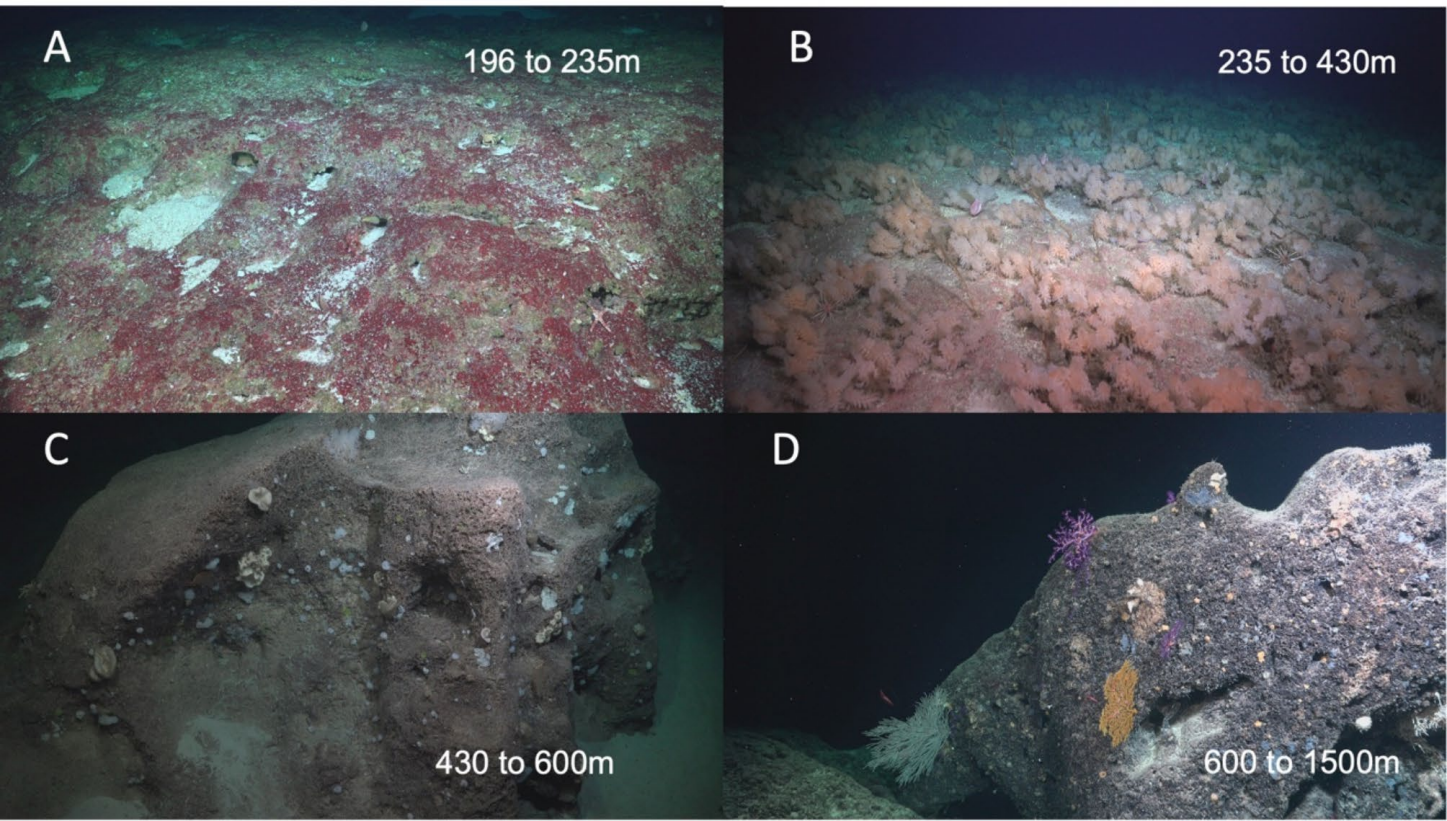
Image plate of example of vertical zonation along the flanks of the seamount. While there were numerous differences between the sides of the feature, four basic zones remained the same on all sides. (**A**) The top of the seamount was predominantly carbonate pavement with sediment filled depressions that hosted cup corals species. CCA commonly encrusted the carbonate (**B**) between 235 and 430 m depth on all side there was a high density community of *Aphrocallistes* cf. *beatrix* sponges with tufts of *Phyllochaetopterus limicolus* worms living amongst the sponges. (**C**) Starting at 430 m down to 600 m, smaller encrusting sponges that were predominantly found on vertical or overhung faces dominated the benthic community. (**D**) the deeper depths (600–1500 m) were a mix of sponges and octocorals taxa made up the comparatively thinly populated patchy community.

While there were differences between the sides of the seamount in terms of abundance at different depths, the overall transition between communities was similar (Figs. 4 and 5). Below 600 m, we found a community that was sparsely populated with octocorals, hexactinellid sponges, and scleractinians making up the majority of the observed individuals (Fig. 5D). At approximately 600 m the community transited to one dominated by encrusting sponge found on vertical walls and over hangs (Fig. 5C). Between 380 and 430 m, depending on flank, the community transited to one with a much higher biomass community dominated by *Aphrocallistes* cf. *beatrix* sponges and *Phyllochaetopterus limicolus* worms with plexaurids and parchment worm (*Chaetopterus* spp.) tubes interspersed (Fig. 5B). This high-density community abruptly ended between 235 and 245 m. Of note, over 70% of the individuals observed had some level of dead tissue, though we did not investigate mortality drivers. *Aphrocallistes* cf. *beatrix* sponge dominated community persisted for approximately 195 m of depth before transitioning to a sparsely populated carbonate pavement with the majority of the sessile fauna being Dendrophylliidae cup corals that were found in sedimented pockets that had collected in pockmarks in the pavement with a significant fish population (Fig. 5A). The start of crustose coralline algae (CCA) was documented between 255 and 265 m on all sides, and continued upwards to the summit (196 m).

**Fig. 6:**
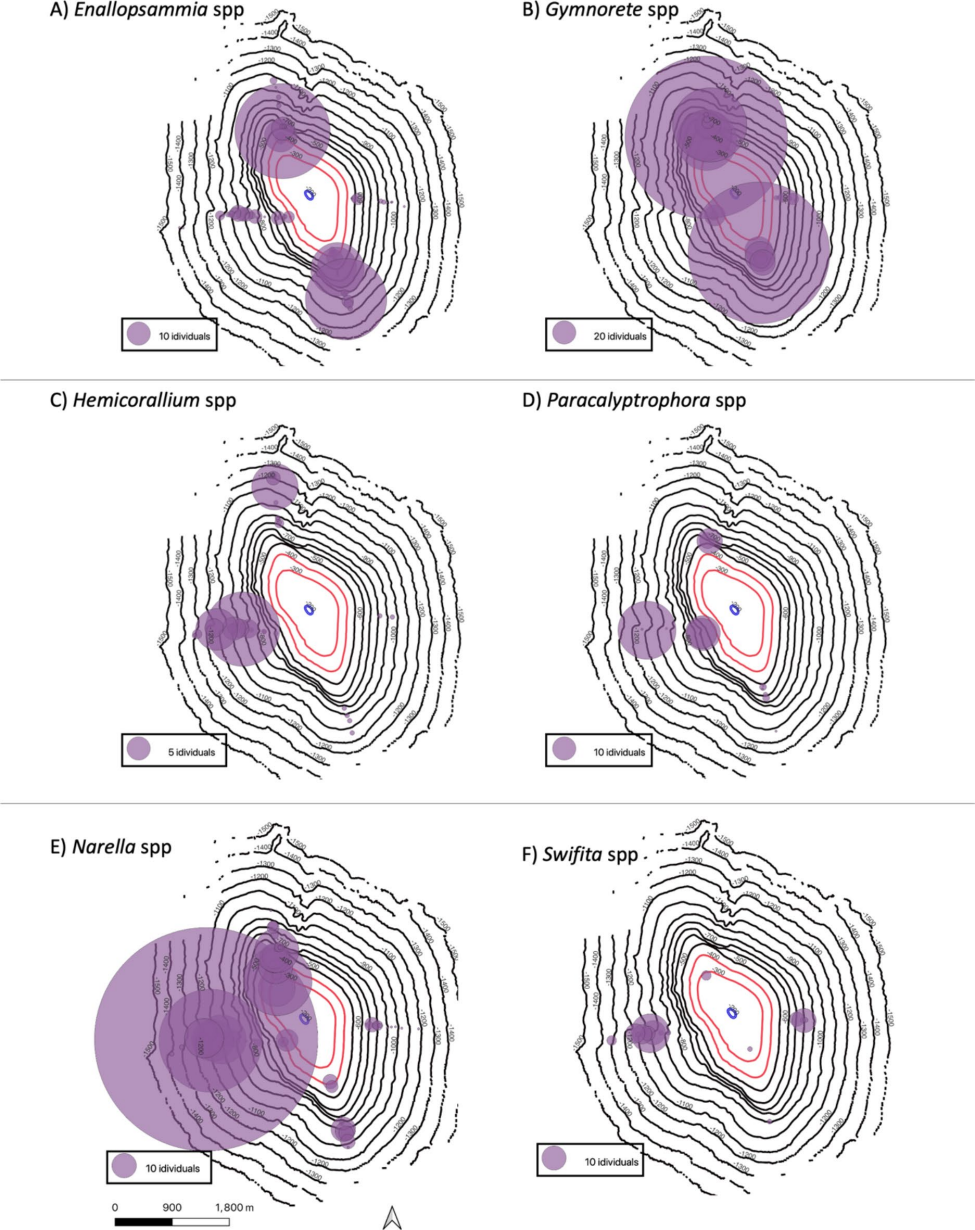
Density of observation of each deepwater taxon by 30 m depth zones. Dots are scaled to the number of individuals seen within each depth bin. Each panel is internally scaled so the dot size to count number is not consistent in all panels.

While the general trend of depth zonation described in the previous paragraph held true for all flanks of the seamount, there were several differences between flanks for specific taxa (Fig. 6A-F). Three distribution patterns were noted across several of the abundant genera. First, certain groups were most prevalent on the North and South flanks of the seamount. *Enallopsammia* and *Gymnorete* spp. were the strongest examples of this pattern (Fig. 6A, B). While these individuals were found on all sides they were far more abundant on the North and South flanks (Supplemental Table 2). Next, certain taxa were found in much higher numbers on both the North and West flanks of the seamount, such as *Hemicorallium*,* Paracalyptrophora*, and *Narella spp.* (Fig. 6C, D, E and Supplemental Table S2). Thirdly, one genus, *Swiftia*, was only found on the East and West flanks (Fig. 6F). However, a one-way ANOSIM comparing the abundance between the different flanks of the seamount indicated that the communities on all sides of the seamount were not significantly different (*p* < 0.01, *R* = 0.069).

**Fig. 7:**
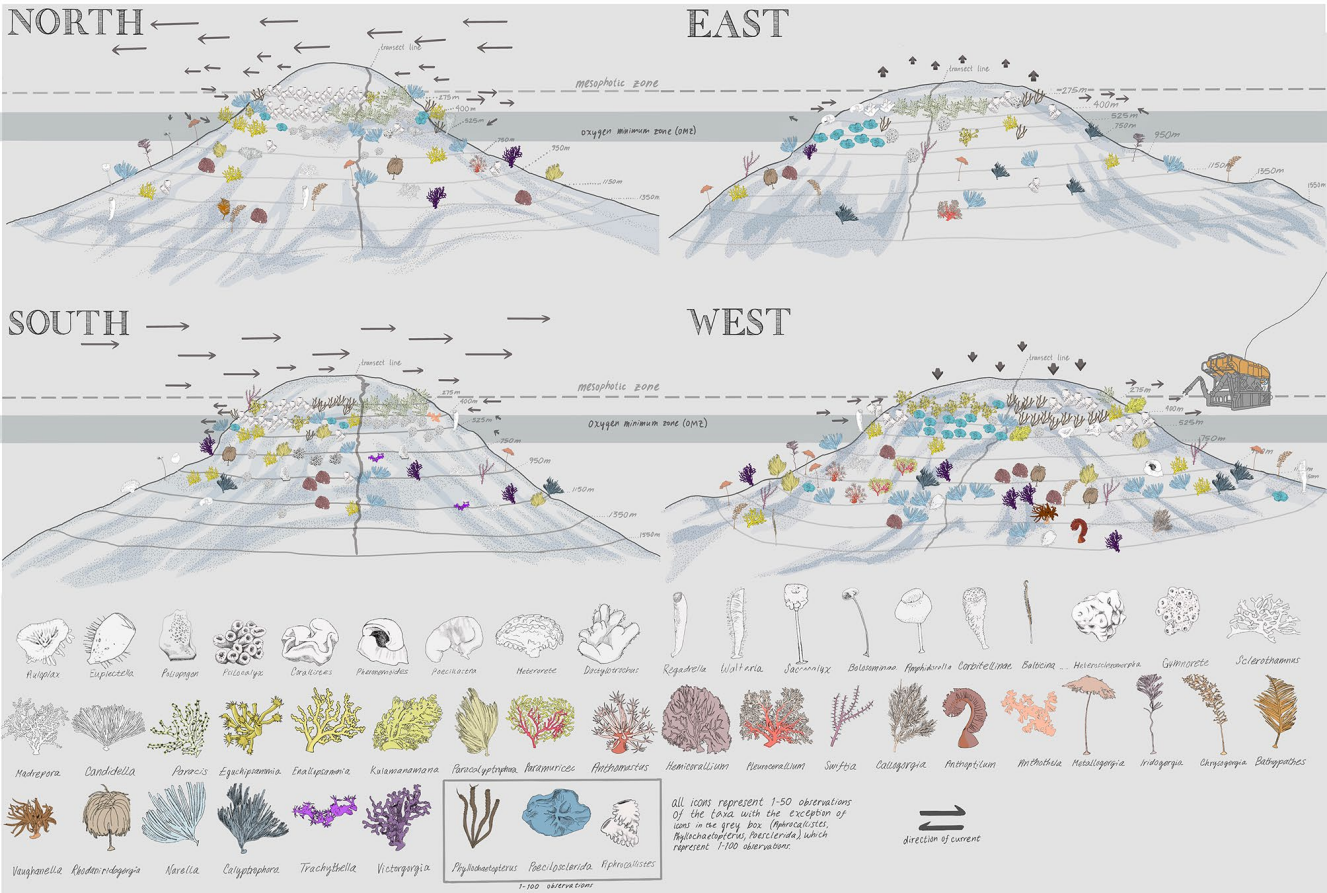
Artist rendition of the zonation of target seamount. In the style of Alexander Von Humboldt and Alexander Keith Johnston, the science party partnered with an artist to make this scientifically accurate artistic rendition of life along the flanks of the seamount. Each panel depicts a flank of the seamount. Each icon represents 50 individuals per 125 m of depth except for the three genera with a black box. They represent 100 individuals. Artist: Constance Sartor, © 2023 CRS. Used by Permission.

The East side of the seamount had the lowest abundance and diversity of sessile fauna (Figs. 4B and 7). The rarefaction curves for the east sides showed a notable reduction in the number of genera observed and the curve had a shallower slope than the other sides indicating that our sampling effort had approached saturation (Supplemental Fig. 1). The curves for the North and South transect are very similar while the West side of the feature had the highest biodiversity and the curve of the slope indicated that there are still additional taxa that were not documented by our level of sampling effort.

The total beta diversity index for the seamount was relatively high (0.831 out of 1). When comparing the local contribution to beta diversity (LCBD) across the flanks, there was only one discernable pattern of flank by depth. The North and South flank depths between 750 and 900 m had an outsized effect on the beta diversity, but otherwise, no single flank or depth appeared to strongly contribute to beta diversity below 300 m. The shallows, however, had strong contributions to beta diversity regardless of flank (Supplemental Figure S2 and Supplemental Table S3).

The role of seafloor slope was a significant predictor of abundance from the generalized mixed methods model (slope: *p* < 0.01, vertical: p = < 0.01, overhang: p = < 0.01) compared to flat areas with less than a 15 deg slope. The role of genus played a significant role in the model with many genera exhibiting a preference for steeper or shallow slopes (Supplementary Table S1).

Opportunistic collections of deep-water benthic megafauna at this seamount provided additional species diversity and biogeographic insights (Supplementary Table S4). Cosmopolitan coral species included those well-represented throughout the US central Pacific^23^ such as *Metallogorgia melanotrichos*, and the colonial scleractinians *Madrepora oculata* and *Enallopsammia rostrata*. Central Pacific bathyal species, including *Paracalyptrophora hawaiiensis*, *Calyptrophora agassizii*, *Narella horrida*, *Narella alata*, *Bebryce brunnea*, *Rhodaniridogorgia superba*, and *Hemicorallium* cf. *imperiale*, were also represented on this seamount and are indicative of biogeographic affinities with the Hawaiian archipelago to the northeast at deeper depths (> 1000 m). A shallower, morphologically-distinct congener of *Metallogorgia melanotrichos*, identified as *Metallogorgia* sp. 4, was also observed at 953 m. The observed distribution patterns of this species to-date throughout the Phoenix Archipelago suggests a narrow geographic distribution within the genus (see *Metallogorgia* sp. 4 in Auscavitch et al.^6^). In addition to the cosmopolitan deep-water stony corals, *M. oculata* and *E. rostrata*, solitary scleractinians and smaller colonial species were also collected across the seamount depth gradient. Among the sponges, the morphological identification of *Aphrocallistes* cf. *beatrix* at this site indicates a substantial expansion of its reported range into the central Pacific from western Indo-Pacific archipelagos and higher southern latitude regions around New Zealand compared to records in the World Register of Marine Species (www.marinespecies.org). In summary, these collections support initial findings by Auscavitch et al.^6^that the Phoenix Archipelago is an area of biogeographic overlap or transition for southern hemisphere, equatorial Indo-Pacific, and north Pacific deep-water corals, but also other habitat-forming organisms like glass sponges (Fig. 5B).

Finally, we worked with an artist (C. Sartor) to visualize all applicable data gathered above in a Von Humboldt-style seamount infographic (Fig. 7). Because visualization of a full seamount cannot be achieved photographically, we used this artistic rendering to conceptualize ecological zonation overlaid with available oceanographic information and abiotic data.

## Discussion

There are many open questions regarding how benthic communities spatially differ across flanks, and how those differences align or correspond with oceanographic variables. We investigated a relatively small and symmetric “model” seamount, and found that on four seamount flanks (one in each cardinal direction), similar communities were present at similar depths within an approximate depth bin of 50 m. However, on a finer scale of depth change (~ 30 m), we observed patchy distribution and relative heterogeneity of taxa between flanks. Within the larger depth bands, we posit four putative types of biological assemblages that were observed on all flanks of the feature^24^, all starting and ending within 50 m of depth on each transect. However, we also observed that abundance within assemblages differed on a taxa-by-taxa basis across the seamount flanks, suggesting flank-specific preferences that likely reflect oceanographic current dynamics. To illustrate this concept, we applied these assemblages to a Von Humboldt-style seamount infographic (Fig. 7) to lay the foundation for ecological zonation work on seamounts and pose new hypotheses for understanding benthic community ecology on seamounts.

While vertical zonation with depth has been well-documented globally, and is typically treated as a continuous linear variable^25^, we have very little context for how that vertical zonation may or may not differ circumferentially around a seamount (but see^15,16,26^). We found four assemblages that were roughly consistent with depth, and were found on all four flanks of the seamount, suggesting that zonation might be relatively robust to variable oceanographic conditions, including temperature, salinity, oxygen, and currents, on this morphologically simple seamount. However, we also observed patchiness within a depth band, which is consistent with many previous observations of hyperlocal habitat preference, for example, on a single boulder or a single face of a vertical wall. Such patchiness has yet to be well-understood, but our results suggest an intermediate driver of habitat preference between depth and benthic substrate. Though we observed hyperlocal patchiness, we observed the same taxa throughout the depth band circumferentially, but not in the same abundance. For example, some taxa (e.g. *Narella* spp. *Paracalyptrophora* spp., and *Hemicorallium* spp., were found in patchy abundance on all flanks of the seamount, but were found in much higher abundances overall on the North and West flanks (Fig. 6). While additional work will be required to attribute a causal relationship to this pattern, the ADCP data down to 600 m indicated strong shifts in current velocities that vary by seamount flank (Fig. 1). Thus, our data demonstrate that vertical zonation patterns are not strictly a function of depth; instead, they are likely a triangulation of depth, benthic substrate, and oceanography^27^. This triangulation is not surprising, as many predictive habitat models use these variables in concert^27^; however, this seamount is the first to empirically demonstrate the circumferential pattern of diversity, coupled with abundance patterns corresponding to oceanography, including temperature, salinity, oxygen, and currents. This suggests that proper management of seamounts - especially seamounts that extend into the mesophotic or photic zone - must incorporate protection of the broader oceanographic conditions that are drivers of their diversity, including surface waters^28^.

There are few previous studies to our knowledge to examine benthic biodiversity circumferentially that had findings that differed from ours in several key aspects. Du Preez et al.^16^ documented nine different types of communities on Cobb Seamount, a large guyot in the North Pacific, but five of those communities were located on the flat top structure of the guyot, and oceanographic variables were not measured and thus not cross-correlated. Similar to our study, however, Du Preez et al.^16^) did find evidence of taxonomic structuring by depth and substrate characteristics such as slope on multiple flanks of the feature. A recent study similarly found taxonomic structuring by depth, slope, and substrate type (bedrock, cobble, pebble, mixed vs. soft sediment, or spicule mat) for sponges in the North Atlantic^29^, suggesting again that the empirical determination of benthic taxa is not determined by depth alone. Morgan et al.^15^identified nine community assemblages from an extensive survey of Mokumanamana (Necker) Island, but found no evidence of circumferential zonation. However, it is important to note that Mokumanana Island is more than 10 times larger than our focal seamount, has a fundamentally different feature shape, and is far more morphologically variable. Seamount shape has recently been suggested as an important factor in driving benthic community structure^13^, possibly due to how shape influences local hydrodynamics. Taken together, these findings demonstrate the importance of multiple surveys on different feature shapes, sizes, and regions in order to more broadly depict biological patterning.

Of all the taxa encountered, *Enallopsammia* spp. corals were the most influenced by oceanographic factors within their habitable depth range, and displayed strong sensitivity to both current and dissolved oxygen concentrations (Figs. 1, 2 and 3). This scleractinian coral was found on all flanks; however it was observed in much greater numbers on the North and South flanks that experienced the most persistent current flow across the depth range sampled (Fig. 1). This is consistent with previous findings such as Tracey et al.^30^ and Hebbeln et al.^31^, which have demonstrated *Enallopsammia* spp. sensitivity to current flow. *Enallopsammia*spp. and the other colonial scleractinians appear to be strongly influenced by dissolved oxygen (DO) concentration and numerous studies have detailed the importance of oxygen levels on coral communities^7,32,33^. In line with previous work, we observed a clear gap in coral coverage where DO levels dropped (Fig. 3)^21,34^. Oxygen minimum zones (OMZ) are geographically widespread water column strata where dissolved oxygen concentrations decline (defined as DO concentrations < 22 µmol/L^7^). Regional differences in the OMZ may result from variation in oceanographic currents, productivity, and respiration by marine organisms^32^. Understanding the effect of oxygen sensitivity will help to interpret the influence of the OMZ on benthic communities, which is predicted to dramatically expand due to climate change^7^, in some cases by as much as eight million cubic kilometers by the end of this century^35^.

Organismal sensitivity and habitat preference contribute to community-level biodiversity, and previous studies have measured beta diversity to compare communities on different seamount sections. Victorero et al.^36^ investigated the beta diversity of Annan Seamount in the north Atlantic and found a beta diversity of 0.92 over a depth range of 200–2700 m, which is similar to our value of 0.831 over a depth range of 196–1500 m, indicating that seamounts even in different ocean basins consistently have a high level of variation across depths. Victorero et al.^36^compared beta diversity of communities parsed along a single transect, whereas we calculated beta diversity on all flanks by depth, and did not find much variation across the seamount even though there were shifts in abundance distributions by taxa (Supplemental Figure S2, Supplemental Table S3). This suggests that, with the exception of the shallowest depths, beta diversity is highly robust within depth ranges by flank. Given the clear patterns of uneven distribution within a depth range, our observations of robust beta diversity are somewhat surprising, and may be an indication of previously unrecognized ecological competition among deep-sea benthic megafauna. This observation raises the question as to whether or not sessile benthic organisms compete for specific, environmentally-optimal patches of seafloor, and therefore may adaptively partition themselves by seamount flank within a depth range to minimize competition. Competition in low biomass coral communities is a novel concept, but may be ecologically plausible given the qualitatively observed clumped dispersion pattern that commonly appears: high density communities are often observed in a small benthic footprint, surrounded by seemingly comparable but uninhabited substrate. As such, though the mechanisms contributing to ideal settlement location are not yet understood, they clearly exist. This concept thus leads to these novel hypotheses:

### Hyp 1

In high biodiversity seamounts, benthic taxa will spatially partition by seamount flank to maximize available resources delivered by variable current flow.

### Hyp 2

In high biodiversity seamounts, benthic taxa will spatially partition by seamount flank to maximize available high quality benthic substrate (geology, slope, etc.)

One factor that may contribute to idealized settlement substrate may be slope steepness, which has long been hypothesized as a factor contributing to successful prey capture via topographically-induced current flow^37^. As such, we examined how slope steepness would impact the abundance of sessile fauna. Nearly half of our taxa (20 out of 45) showed a statistically significant preference for at least one of the four categories of slope we identified (flat, sloped, vertical, and overhung)(Supplemental Table S1). Previous work has well established that seafloor steepness plays an important role in habitat preference for sessile filter/suspension feeding benthic organisms^27,38^. However, we noted a novel biotope - a physical habitat associated with a particular ecological community^39^. Specifically, the underside of rocky overhangs, or hardgrounds with slopes in excess of 90 degrees. We found nearly all of the *Poecillastra* and *Autoplax* spp. sponges in these overhanging environments, while several other taxa like *Psilocalyx* and *Gymnorete* spp. were found in high abundance on both vertical and overhang environments. One advantage of inhabiting the underside of overhangs may be changes in hyperlocal hydrodynamics, which may in turn modulate current speed, eddy dynamics, or sedimentation that may impact feeding success and/ or settlement probability.

Taken together, the high habitat heterogeneity of seamounts prevents easy extrapolation of biodiversity information from bathymetrically or geographically narrow surveys, thus justifying a more comprehensive approach to seamount characterization. Our results suggest that a single survey, surveys on a single side, or surveys that are limited to a narrow depth range on a seamount will not adequately describe the entire feature. This is not surprising, given that there are already numerous oceanographic demonstrations of seamount heterogeneity, for example large bathymetric gradients in temperature^40^and dissolved oxygen concentrations^7^. Though the justification for increased survey effort on seamounts is apparent, very few seamounts have been sampled or visited more than once or twice, though there are a few notable exceptions such as Davidson and Axial seamounts^41,42^. However, recent exploratory efforts (e.g., NOAA Office of Ocean Exploration^43^, the Ocean Exploration Trust^44^, Schmidt Ocean Institute^45^and others) are amassing publicly available seamount data and biological collections that may be approaching a data density useful for addressing these types of questions. Data collected across previous expeditions may become urgently necessary as regulations are being written for environmental impact statements that will be required for deep-sea mining and bottom trawling on seamounts, without comprehensive data available^46^. As additional research illuminates the complexities of these seamounts and their interactions with the ocean around them (e.g. Mashayek et al.^47^), proper management will need to span an area large enough to adequately incorporate oceanographic conditions while also managing current and future threats from exploitation of resources within this delicately interconnected system. For example, Leitner et al.^48^suggests that seamounts interact with oceanographic surface dynamics via chlorophyll enhancements, which benefits fish density and biomass in commercially-relevant fisheries. The science describing how seamounts influence deep-ocean upwelling is nascent, but is emerging as a critically important role for how seamounts influence open ocean dynamics^47,49^; our study highlights the potential for these mixing and surface dynamics to similarly influence benthic communities on seamount slopes.

Starting with HMS Challenger, the efforts to explore and characterize the deep ocean have been steadily increasing for over a century and a half, but still have not kept pace with the rate of exploitation^50^. Now, with more than eight billion people on Earth, we are simultaneously exploiting (fisheries, mining, etc.) and protecting (e.g. 30 × 30) the deep ocean at unprecedented rates^51^. Humanity is faced with the need to make policy decisions about how to manage our deep sea resources with a limited understanding of deep-sea biology when compared to the state of the science in terrestrial ecosystems^52^. Seamounts are particularly important in the open ocean because they make up a substantial portion of the hard ground benthic substrate shallower than 3000 m away from the continental slopes and host a wide range of life, serving as both a way station for migrating species^9^and habitat for numerous endemic species^11^. However, all seamounts are not the same, and deep-sea ecologists have only a limited understanding of different biological communities on a single seamount. Terrestrial infographics like the Von Humboldt mountains have become iconic and have withstood the test of time because they created a compelling, simple visual to advance an important idea: that elevation and wind direction on terrestrial mountains could shape biological communities. Despite the intense interest in oceans at large and the deep sea in particular, we have yet to rally around the nuance and biological complexity of seamounts because they remain dark, hidden, and opaque. Our intent is to emulate the stylings of Von Humboldt at this pivotal moment in ocean history - both for conservation and exploitation - as a rallying point to showcase the beauty, nuance, and complexity of seamounts, and how much more we could know.

## Methods

### Site selection

**Fig. 8:**
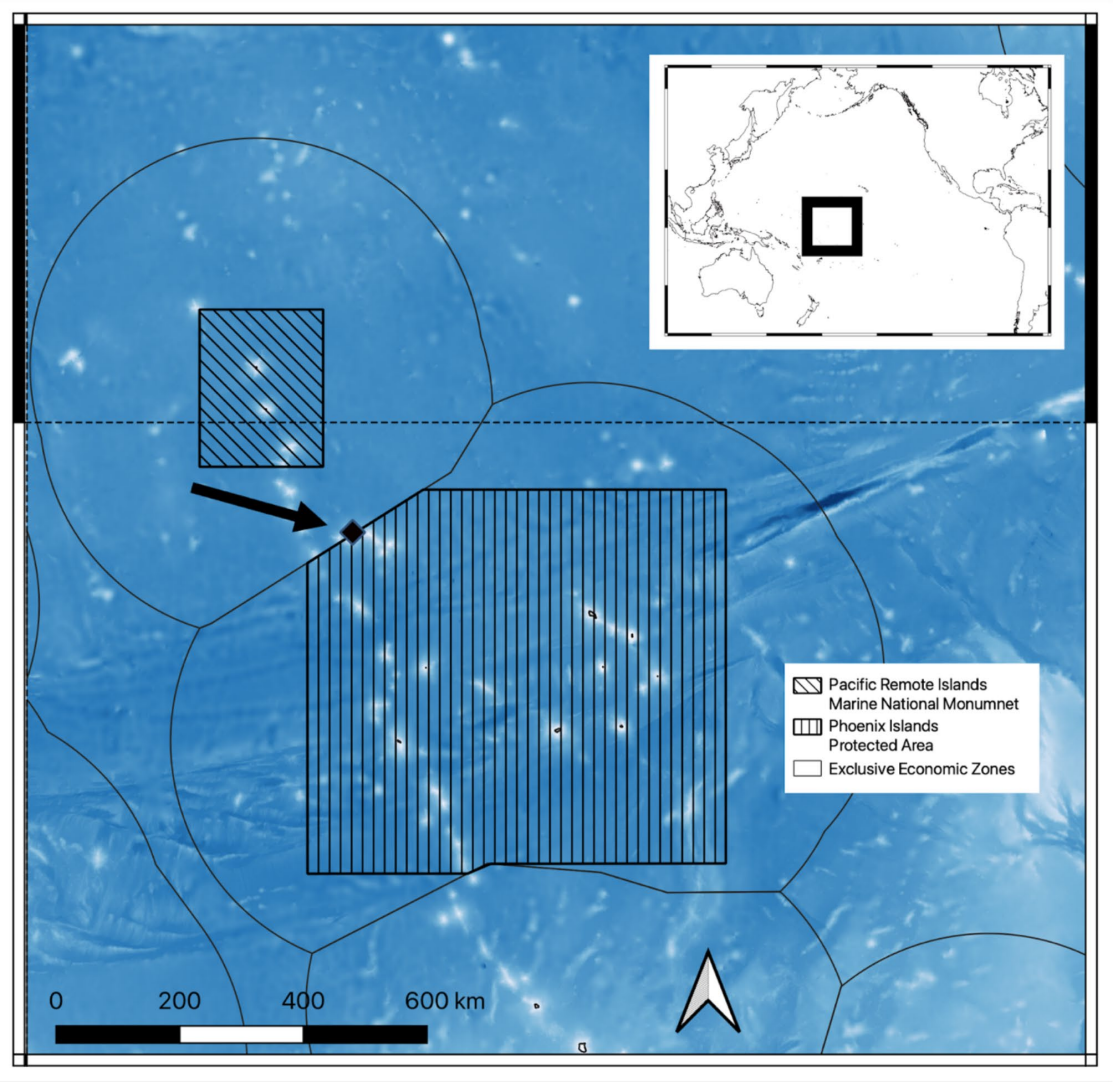
Map of study area. The focus of this project is a single seamount feature that is part of a cluster of seamounts that is being referred to as “The Winslow Complex” in the Phoenix Archipelago. This seamount is just south of the Equator (dashed line) in US territorial waters, surrounding the Howland and Baker portion of the Pacific Islands Heritage Marine National Monument. The feature rises from 5,000 m depth to just shallower than 200 m. The features’ location on the equator makes for dynamic oceanographic conditions with high current velocities with the equatorial undercurrent hitting the top of the feature. Map made with QGIS version 3.34.

All work was conducted on a currently unnamed seamount within United States (US) waters, in the area surrounding the Howland and Baker National Marine Monument, near the border with Kiribati’s exclusive economic zone (EEZ) (1.61 S, 175.20 W, Fig. 8). This seamount rises from a depth of more than 5,000 m up to 196 m (into the mesophotic zone) and is roughly symmetrical at 20-meter bathymetry resolution, with even contour lines and similar depths and slopes on each flank. The only major bathymetric variation is the remnants of a discrete mass wasting event on the North side of the feature. The water around the target feature is heavily influenced by a complex system of east- and west-flowing currents and countercurrents that include the Equatorial Undercurrent (EUC), North Equatorial Countercurrent (NECC), North and South Equatorial Currents (NEC and SEC, respectively), and the North and South Deep Countercurrents (NDCC and SDCC respectively)^20^. This feature lies in close proximity to the Winslow Reef Complex, which has been noted for its high benthic invertebrate abundance^21^and contains four other seamounts (within 30 NM) with one peak reaching as shallow as 20 m, supporting a robust photic coral reef^47^. The target seamount is in US waters only 3 km North of the border of Kiribati’s EEZ surrounding the Phoenix Islands and 120 km south east of the boundary of the Howland and Baker Unit of the Pacific Islands Heritage Marine National Monument. While this feature is not inside of a marine protected area, its proximity to one of the largest deep-water marine protected areas in the world (the PRI), and the proximity to the formerly-protected Phoenix Islands Protected Area in Kiribati, makes it a particularly interesting study site because of potential spillover effect and the implication of regional marine policy (or lack thereof). This unnamed seamount was newly discovered onboard a Schmidt Ocean Expedition expedition to the US Phoenix Islands archipelago, aboard the *R/V Falkor* expedition FK210605 in June 2021.

### Data collection and annotation

In June 2021, four continuous transects were conducted during four dives (S0434, S0435, S0436, and S0437), using Schmidt Ocean Institute’s SuBastian remotely operated vehicle (ROV) aboard the *R/V Falkor*. Each transect was conducted on a single flank of the seamount based on cardinal direction (N, S, E, W), all starting between 1200 and 1500 m and terminating at the summit (196 m). The average transect bottom track length was 2250 m. All surveys were conducted from deep to shallow, perpendicular to the bathymetric contours, typically at an over-ground speed of 0.25–0.5 kt (0.1–0.25 m/s), stopping only to zoom into features of interest or to collect physical samples. SuBastian was equipped with an onboard CTD sensor (SeaBird FastCAT SBE49) and oxygen optode (Aandera O2 optode 4831), which logged continuously. Seafloor position was logged using an ultrashort baseline (USBL) navigation sensor (Sonardyne WMT 6G) and an inertial guidance system (Sonardyne Sprint). During the end of dive S0433, the CTD data was intermittent, and was unavailable for dives S0434, S0435, and S0436. To substitute for these gaps in CTD data, two shipboard CTD casts (SBE 911+) were conducted immediately following dives S0434 and S0435 in waters approximately 2,000 m deep in close proximity to the start of the ROV dive. Data from shipboard CTDs were processed using Seabird Data Processing v7.26.7. ROV CTD data were processed by the crew of *R/V Falkor* using the NOAA created Scientific Computing System (SCS) (https://scsshore.noaa.gov/). Data from both sensors were combined and analyzed using Ocean Data View (odv.awi.de).

Current data was collected by R/V Falkor’s Teledyne Ocean Surveyor 75 kHz Acoustic Doppler Current Profiler (ADCP) and the data were processed by the University of Hawaii Common Ocean Data Access System (www.currents.soest.hawaii.edu/uhdas_home/). The processed data yielded current data in 25-meter depth bins and 5-minute temporal ensembles. ADCP current data vector components were converted to cardinal direction and velocity using R (www.r-project.org) then the data was visualized in QGIS (www.qgis.org).

Bathymetry was collected by R/V Falkor’s two multibeam sonar systems Kongsberg EM710 (Freq) and EM302 (30 kHz). Data were processed onboard using a Fledermaus Qymera workflow (qps.com). A cube-processing algorithm was applied to clean the sonar data. No tidal corrections were performed given the water depth. Sonar data was gridded at 20 m.

The seafloor video was annotated from the time the ROV reached the sea floor until it left the bottom as long as the ROV was in visual contact with the bottom ( < ~ 2 m altitude). All corals, sponges, and other dominant sessile benthic fauna greater than 5 cm in size were recorded. All annotated organisms were identified to the lowest taxonomic level possible from the video utilizing the physical specimens that were collected to aid the identification whenever possible (Supplementary Table S4). Organisms that could not be identified to the species level were instead assigned a morphospecies identifier. Additional taxonomic literature and taxonomic expertise was consulted for specimen collection identification. To determine if slope had an effect on habitat preference, we visually estimated the angle of the seafloor for each sessile organism observation. Seafloor slope was categorized for the location of the coral or sponge attachment location in one of four categories: flat (< 15 deg), slope (15–70 deg), vertical (70–90 deg), or overhang (> 90 deg).

### Statistical analysis

All statistical analyses were conducted using R. In order to test for significant differences between the sides of the seamounts, Analysis of Similarities (ANOSIM) were conducted using morphospecies binned by 30-meter depth zones. Using the same depth zone binning, we also created rarefaction curves for each side of the feature (Supplemental Fig. 1). The ANOSIM and species accumulation curves were both made using the R package ‘vegan’^48^.

To investigate the role of slope on abundance of sessile organisms, we conducted a generalized linear mixed methods model with slope and genus as covariates using the ‘glmer.nb’ function from the ‘lm4’ package^49^. We employed an additive model selection process comparing Akaike information criterion (AIC) scores to choose the best model^50^. We employed a negative binomial distribution for best fit; we could not use a Poisson distribution because it underestimated the number of zeros in our dataset.

Total beta diversity and Local contribution to beta diversity (LCBD) was calculated for 30-meter depth bins using the ‘beta.div’ function from the ‘adespatial’ package^51^. For this analysis, we removed all the singletons from the dataset because they were not deemed to be common representatives of the biological community. The LCBD represents the ecological comparative uniqueness of each sample. The significance for each bin was assessed through permutation analysis (999 iterations), testing the null hypothesis that genera distribution is random among the sampling depths. The methods behind this approach are outlined by Dray, et al.^51^. All methods were carried out in accordance with relevant guidelines and regulations.

## Electronic supplementary material

Below is the link to the electronic supplementary material.

Supplementary Material 1

## Data Availability

Datasets collected by R/V Falkor during this expedition (FK210605) are available through the R-to-R program (https://www.rvdata.us/search/cruise/FK210605). Video annotations are available through Zenodo (https://zenodo.org/records/13255648), Physical specimens are housed at either the Invertebrate Zoology Collections at the Museum of Comparative Zoology at Harvard University or are available upon request form the Rotjan Lab at Boston University (BU-RL). See Supplementary Table S4 for repository Identification numbers.

## Declarations

### Dual publication

Much of this manuscript was part of BK’s Boston University dissertation (https://hdl.handle.net/2144/47411). This does not constitute dual publication given Natures guidelines on Pre-publicity “Material in a paper submitted to Scientific Reports may also have been published as part of a PhD or other academic thesis.”
