# Supplementary material for: Multi-faceted examination of a deepwater seamount reveals ecological patterns among coral and sponge communities in the equatorial Pacific
Source: Sci Rep. 2025 Jan 17;15:2270. doi: 10.1038/s41598-025-86163-z (PMC11742039; doi:10.1038/s41598-025-86163-z)
Supplement: Supplementary file 1 — Supplementary Material 1 [file 41598_2025_86163_MOESM1_ESM.docx]

# Title: Multi-faceted examination of a deepwater seamount reveals ecological patterns among coral and sponge communities in the equatorial Pacific

# Supplementary information

Authors: Brian RC Kennedy ^1^, Steven Auscavitch^1^, Timothy Shank^2^, Constance Sartor^3^, Anameere Tennaba^4^, Alexis Weinnig^5^, Randi D. Rotjan^1, 6^

Affiliations:

1. Department of Biology, Boston University Boston MA USA
2. Biology Department, Woods Hole Ocean Oceanographic Institution, Woods Hole MA USA
3. University of Guam, Mangilao GU USA
4. GeoScience Division ECOP Office, Ministry of Fisheries and Marine Resources Development, Republic of Kiribati, Tarawa, Kiribati
5. Eastern Ecological Science Center, United States Geological Survey, Kearnesville, WV USA
6. Blue Nature Alliance, Arlington, VA, USA

Key words: Seamount, Pacific Islands Heritage, Deep-sea coral, Vertical zonation, Deep-sea sponges, community composition


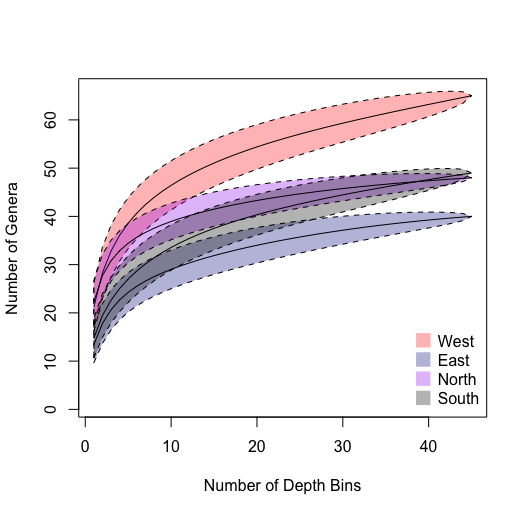


**Supplemental Figure S1.** Rarefaction curves by 30-meter depth bin for each side of the seamount. The North and East dives are nearly identical. There is a noticeable difference between the East and West dives. The East side of the feature has a much lower number of unique taxa and approaches the asymptote much quicker than the north side which has a larger total number of taxa retains a steeper slope throughout the sampling range.


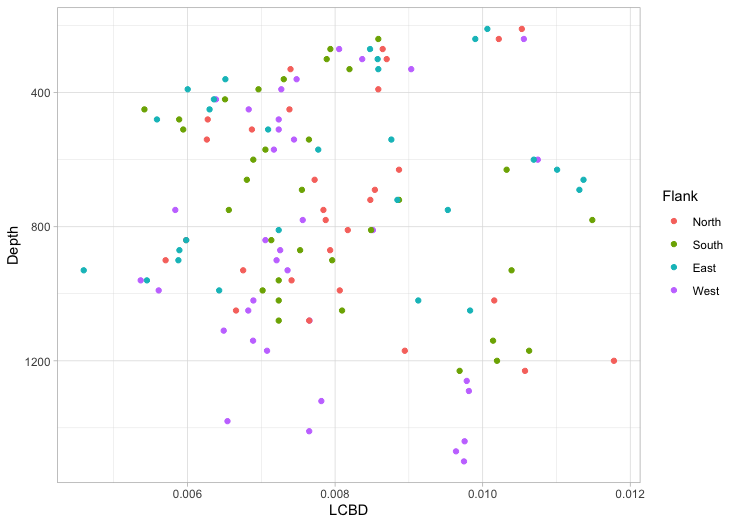


**Supplemental Figure S2.** Local Contribution to Beta Diversity (LCBD) for each side of the seamount. The LCBD represents the ecological comparative uniqueness of each sample. The wide variation of values between each side of the feature at any given depth indicates that the communities in those depths contribute differently to the overall beta diversity of the feature and that each side is different. See Supplemental table 3 for the statistical significance of each depth bin.

##### Supplemental Table S1.

Percent of individuals observed via each slope category.

| Genus | flat (<15 deg) | slope (15-70deg) | vertical (70-90 deg) | Overhang (>90 deg) |
| --- | --- | --- | --- | --- |
| *Aphrocallistes* | 54.04 | 40.92 | 3.35 | 1.70 |
| *Phyllochaetopterus* | 3.56 | 94.09 | 1.99 | 0.37 |
| *Paracis* | 28.77 | 69.71 | 0.00 | 1.52 |
| *Gymnorete* | 1.28 | 12.16 | 63.36 | 23.20 |
| *Narella* | 15.33 | 17.92 | 42.69 | 24.06 |
| *Enallopsammia* | 24.46 | 36.69 | 30.22 | 8.63 |
| *Eguchipsammia* | 14.55 | 83.18 | 1.82 | 0.45 |
| *Paracalyptrophora* | 11.63 | 20.93 | 37.21 | 30.23 |
| *Metallogorgia* | 26.83 | 69.51 | 3.66 | 0.00 |
| *Hemicorallium* | 1.30 | 18.18 | 42.86 | 37.66 |
| *Victorgorgia* | 7.94 | 22.22 | 33.33 | 36.51 |
| *Psilocalyx* | 0.00 | 0.00 | 59.26 | 40.74 |
| *Calyptrophora* | 2.22 | 31.11 | 15.56 | 51.11 |
| *Madrepora* | 2.22 | 35.56 | 20.00 | 42.22 |
| *Swiftia* | 6.98 | 25.58 | 34.88 | 32.56 |
| *Dactylotrochus* | 0.00 | 55.17 | 44.83 | 0.00 |
| *Poecillastra* | 0.00 | 7.41 | 0.00 | 92.59 |
| *Chrysogorgia* | 8.00 | 72.00 | 12.00 | 8.00 |
| *Anthomastus* | 0.00 | 0.00 | 58.33 | 41.67 |

##### Supplemental Table S2.

Counts of individuals per depth bin for each flank of the seamount for each of the 25 most abundant taxa.

| **Genus** | **Flank** | **275 m** | **400 m** | **525 m** | **650 m** | **775 m** | **900 m** | **1025 m** | **1150 m** | **1275 m** | **1400 m** | **1525 m m** |
| --- | --- | --- | --- | --- | --- | --- | --- | --- | --- | --- | --- | --- |
| ***Anthomastus*** | **West** | **0** | **0** | **0** | **0** | **0** | **2** | **8** | **1** | **0** | **0** | **0** |
| ***Anthomastus*** | **East** | **0** | **0** | **0** | **0** | **0** | **0** | **0** | **0** | **0** | **0** | **0** |
| ***Anthomastus*** | **North** | **0** | **0** | **0** | **0** | **0** | **13** | **0** | **0** | **0** | **0** | **0** |
| ***Anthomastus*** | **South** | **0** | **0** | **0** | **0** | **0** | **0** | **0** | **0** | **0** | **0** | **0** |
| ***Aphrocallistes*** | **West** | **366** | **278** | **3** | **4** | **0** | **0** | **0** | **0** | **0** | **0** | **0** |
| ***Aphrocallistes*** | **East** | **156** | **46** | **0** | **0** | **0** | **0** | **0** | **1** | **0** | **0** | **0** |
| ***Aphrocallistes*** | **North** | **1406** | **1070** | **6** | **9** | **4** | **2** | **1** | **0** | **0** | **0** | **0** |
| ***Aphrocallistes*** | **South** | **613** | **504** | **10** | **0** | **0** | **1** | **0** | **0** | **0** | **0** | **0** |
| ***Auloplax*** | **West** | **0** | **0** | **17** | **0** | **0** | **0** | **0** | **0** | **0** | **0** | **0** |
| ***Auloplax*** | **East** | **0** | **0** | **0** | **0** | **0** | **0** | **0** | **0** | **0** | **0** | **0** |
| ***Auloplax*** | **North** | **0** | **0** | **0** | **0** | **0** | **0** | **0** | **0** | **0** | **0** | **0** |
| ***Auloplax*** | **South** | **0** | **0** | **0** | **0** | **0** | **0** | **0** | **0** | **0** | **0** | **0** |
| ***Bolosominae*** | **West** | **0** | **0** | **0** | **0** | **0** | **0** | **0** | **0** | **0** | **0** | **0** |
| ***Bolosominae*** | **East** | **0** | **0** | **0** | **0** | **0** | **0** | **0** | **0** | **0** | **0** | **0** |
| ***Bolosominae*** | **North** | **0** | **0** | **0** | **0** | **1** | **3** | **1** | **0** | **0** | **0** | **0** |
| ***Bolosominae*** | **South** | **0** | **0** | **0** | **0** | **0** | **1** | **0** | **0** | **0** | **0** | **0** |
| ***Calyptrophora*** | **West** | **0** | **0** | **0** | **0** | **0** | **1** | **4** | **23** | **0** | **0** | **0** |
| ***Calyptrophora*** | **East** | **0** | **0** | **0** | **0** | **3** | **0** | **1** | **0** | **2** | **0** | **0** |
| ***Calyptrophora*** | **North** | **0** | **0** | **0** | **0** | **0** | **0** | **0** | **0** | **0** | **0** | **0** |
| ***Calyptrophora*** | **South** | **0** | **0** | **0** | **0** | **0** | **10** | **1** | **0** | **0** | **0** | **0** |
| ***Candidella*** | **West** | **0** | **0** | **0** | **0** | **0** | **0** | **0** | **0** | **0** | **0** | **0** |
| ***Candidella*** | **East** | **0** | **0** | **0** | **0** | **0** | **0** | **0** | **0** | **0** | **0** | **0** |
| ***Candidella*** | **North** | **0** | **0** | **3** | **16** | **0** | **0** | **0** | **0** | **0** | **0** | **0** |
| ***Candidella*** | **South** | **0** | **0** | **0** | **0** | **0** | **0** | **0** | **0** | **0** | **0** | **0** |
| ***Chrysogorgia*** | **West** | **0** | **0** | **0** | **0** | **0** | **0** | **2** | **3** | **7** | **2** | **5** |
| ***Chrysogorgia*** | **East** | **0** | **0** | **0** | **0** | **0** | **0** | **1** | **1** | **1** | **0** | **0** |
| ***Chrysogorgia*** | **North** | **0** | **0** | **0** | **0** | **0** | **0** | **0** | **1** | **2** | **0** | **0** |
| ***Chrysogorgia*** | **South** | **0** | **0** | **0** | **0** | **0** | **0** | **0** | **0** | **0** | **0** | **0** |
| ***Dactylotrochus*** | **West** | **0** | **0** | **0** | **0** | **0** | **0** | **0** | **0** | **0** | **0** | **0** |
| ***Dactylotrochus*** | **East** | **29** | **0** | **0** | **0** | **0** | **0** | **0** | **0** | **0** | **0** | **0** |
| ***Dactylotrochus*** | **North** | **0** | **0** | **0** | **0** | **0** | **0** | **0** | **0** | **0** | **0** | **0** |
| ***Dactylotrochus*** | **South** | **0** | **0** | **0** | **0** | **0** | **0** | **0** | **0** | **0** | **0** | **0** |
| ***Eguchipsammia*** | **West** | **182** | **0** | **0** | **0** | **0** | **0** | **0** | **0** | **0** | **0** | **0** |
| ***Eguchipsammia*** | **East** | **0** | **0** | **1** | **0** | **0** | **0** | **0** | **0** | **0** | **0** | **0** |
| ***Eguchipsammia*** | **North** | **24** | **8** | **0** | **0** | **0** | **0** | **0** | **0** | **0** | **0** | **0** |
| ***Eguchipsammia*** | **South** | **1** | **4** | **0** | **0** | **0** | **0** | **0** | **0** | **0** | **0** | **0** |
| ***Enallopsammia*** | **West** | **0** | **1** | **12** | **5** | **1** | **16** | **22** | **6** | **0** | **1** | **1** |
| ***Enallopsammia*** | **East** | **0** | **0** | **8** | **2** | **0** | **5** | **2** | **1** | **0** | **0** | **0** |
| ***Enallopsammia*** | **North** | **0** | **2** | **70** | **4** | **0** | **3** | **3** | **4** | **0** | **0** | **0** |
| ***Enallopsammia*** | **South** | **0** | **7** | **49** | **1** | **0** | **41** | **11** | **0** | **0** | **0** | **0** |
| ***Gymnorete*** | **West** | **0** | **2** | **24** | **0** | **0** | **0** | **0** | **0** | **0** | **0** | **0** |
| ***Gymnorete*** | **East** | **0** | **1** | **13** | **4** | **9** | **0** | **0** | **0** | **0** | **0** | **0** |
| ***Gymnorete*** | **North** | **0** | **112** | **167** | **66** | **19** | **0** | **1** | **0** | **0** | **0** | **0** |
| ***Gymnorete*** | **South** | **0** | **153** | **52** | **0** | **1** | **0** | **1** | **0** | **0** | **0** | **0** |
| ***Hemicorallium*** | **West** | **0** | **0** | **0** | **1** | **2** | **5** | **27** | **13** | **2** | **1** | **0** |
| ***Hemicorallium*** | **East** | **0** | **0** | **0** | **0** | **0** | **1** | **1** | **0** | **0** | **0** | **0** |
| ***Hemicorallium*** | **North** | **0** | **0** | **0** | **0** | **0** | **5** | **1** | **10** | **4** | **0** | **0** |
| ***Hemicorallium*** | **South** | **0** | **0** | **0** | **0** | **0** | **1** | **2** | **1** | **0** | **0** | **0** |
| ***Madrepora*** | **West** | **0** | **0** | **0** | **0** | **0** | **2** | **34** | **0** | **0** | **0** | **0** |
| ***Madrepora*** | **East** | **0** | **0** | **0** | **0** | **0** | **0** | **0** | **0** | **0** | **0** | **0** |
| ***Madrepora*** | **North** | **0** | **0** | **0** | **6** | **0** | **0** | **3** | **0** | **0** | **0** | **0** |
| ***Madrepora*** | **South** | **0** | **0** | **0** | **0** | **0** | **0** | **0** | **0** | **0** | **0** | **0** |
| ***Metallogorgia*** | **West** | **0** | **0** | **0** | **0** | **1** | **34** | **21** | **0** | **0** | **1** | **4** |
| ***Metallogorgia*** | **East** | **0** | **0** | **0** | **0** | **1** | **4** | **2** | **0** | **0** | **0** | **0** |
| ***Metallogorgia*** | **North** | **0** | **0** | **0** | **0** | **1** | **8** | **0** | **0** | **0** | **0** | **0** |
| ***Metallogorgia*** | **South** | **0** | **0** | **0** | **0** | **0** | **3** | **2** | **0** | **0** | **0** | **0** |
| ***Narella*** | **West** | **0** | **10** | **4** | **1** | **4** | **1** | **36** | **171** | **0** | **4** | **0** |
| ***Narella*** | **East** | **0** | **0** | **0** | **0** | **12** | **8** | **3** | **0** | **1** | **0** | **0** |
| ***Narella*** | **North** | **22** | **39** | **42** | **7** | **3** | **9** | **1** | **0** | **0** | **0** | **0** |
| ***Narella*** | **South** | **0** | **6** | **5** | **0** | **1** | **13** | **21** | **0** | **0** | **0** | **0** |
| ***Paracalyptrophora*** | **West** | **0** | **0** | **0** | **29** | **0** | **0** | **1** | **26** | **0** | **0** | **0** |
| ***Paracalyptrophora*** | **East** | **0** | **0** | **0** | **0** | **0** | **0** | **0** | **0** | **0** | **0** | **0** |
| ***Paracalyptrophora*** | **North** | **0** | **0** | **0** | **18** | **0** | **0** | **1** | **0** | **0** | **0** | **0** |
| ***Paracalyptrophora*** | **South** | **0** | **0** | **4** | **6** | **0** | **0** | **1** | **0** | **0** | **0** | **0** |
| ***Paracis*** | **West** | **21** | **0** | **0** | **0** | **0** | **0** | **0** | **0** | **0** | **0** | **0** |
| ***Paracis*** | **East** | **336** | **0** | **0** | **0** | **0** | **0** | **0** | **0** | **0** | **0** | **0** |
| ***Paracis*** | **North** | **80** | **157** | **0** | **0** | **0** | **0** | **0** | **0** | **0** | **0** | **0** |
| ***Paracis*** | **South** | **195** | **0** | **0** | **0** | **0** | **0** | **0** | **0** | **0** | **0** | **0** |
| ***Phyllochaetopterus*** | **West** | **372** | **795** | **0** | **0** | **0** | **0** | **0** | **0** | **0** | **0** | **0** |
| ***Phyllochaetopterus*** | **East** | **186** | **1** | **14** | **1** | **0** | **0** | **0** | **0** | **0** | **0** | **0** |
| ***Phyllochaetopterus*** | **North** | **10** | **10** | **90** | **0** | **0** | **0** | **0** | **0** | **0** | **0** | **0** |
| ***Phyllochaetopterus*** | **South** | **585** | **19** | **82** | **0** | **0** | **0** | **0** | **0** | **0** | **0** | **0** |
| ***Poecillastra*** | **West** | **0** | **0** | **0** | **0** | **0** | **0** | **0** | **0** | **0** | **0** | **0** |
| ***Poecillastra*** | **East** | **12** | **14** | **0** | **0** | **0** | **0** | **0** | **0** | **0** | **0** | **0** |
| ***Poecillastra*** | **North** | **0** | **0** | **0** | **0** | **0** | **0** | **0** | **0** | **0** | **0** | **0** |
| ***Poecillastra*** | **South** | **0** | **0** | **0** | **0** | **0** | **0** | **0** | **1** | **0** | **0** | **0** |
| ***Psilocalyx*** | **West** | **0** | **0** | **0** | **0** | **0** | **0** | **0** | **0** | **0** | **0** | **0** |
| ***Psilocalyx*** | **East** | **0** | **0** | **0** | **0** | **0** | **0** | **0** | **0** | **0** | **0** | **0** |
| ***Psilocalyx*** | **North** | **0** | **0** | **0** | **0** | **0** | **0** | **0** | **0** | **0** | **0** | **0** |
| ***Psilocalyx*** | **South** | **0** | **0** | **0** | **41** | **13** | **0** | **0** | **0** | **0** | **0** | **0** |
| ***Regadrella*** | **West** | **0** | **0** | **2** | **1** | **0** | **0** | **0** | **0** | **0** | **0** | **0** |
| ***Regadrella*** | **East** | **0** | **0** | **0** | **0** | **0** | **0** | **0** | **0** | **0** | **0** | **0** |
| ***Regadrella*** | **North** | **0** | **0** | **0** | **0** | **0** | **0** | **0** | **2** | **1** | **0** | **0** |
| ***Regadrella*** | **South** | **0** | **0** | **1** | **0** | **0** | **0** | **0** | **0** | **0** | **0** | **0** |
| ***Rhodaniridogorgia*** | **West** | **0** | **0** | **0** | **0** | **0** | **1** | **0** | **0** | **0** | **0** | **0** |
| ***Rhodaniridogorgia*** | **East** | **0** | **0** | **0** | **0** | **0** | **2** | **0** | **0** | **0** | **0** | **0** |
| ***Rhodaniridogorgia*** | **North** | **0** | **0** | **0** | **0** | **0** | **2** | **0** | **0** | **0** | **0** | **0** |
| ***Rhodaniridogorgia*** | **South** | **0** | **0** | **0** | **0** | **1** | **0** | **0** | **0** | **0** | **0** | **0** |
| ***Sclerothamnus*** | **West** | **0** | **0** | **0** | **0** | **0** | **0** | **0** | **0** | **0** | **0** | **0** |
| ***Sclerothamnus*** | **East** | **0** | **0** | **0** | **0** | **0** | **0** | **0** | **0** | **0** | **0** | **0** |
| ***Sclerothamnus*** | **North** | **0** | **0** | **0** | **0** | **0** | **1** | **0** | **0** | **0** | **0** | **0** |
| ***Sclerothamnus*** | **South** | **0** | **0** | **0** | **0** | **0** | **2** | **5** | **2** | **0** | **0** | **0** |
| ***Swiftia*** | **West** | **0** | **0** | **0** | **0** | **0** | **1** | **14** | **12** | **0** | **2** | **0** |
| ***Swiftia*** | **East** | **0** | **0** | **0** | **0** | **3** | **7** | **0** | **0** | **0** | **0** | **0** |
| ***Swiftia*** | **North** | **0** | **2** | **0** | **0** | **0** | **0** | **0** | **0** | **0** | **0** | **0** |
| ***Swiftia*** | **South** | **1** | **0** | **0** | **0** | **0** | **1** | **0** | **0** | **0** | **0** | **0** |
| ***Victorgorgia*** | **West** | **0** | **0** | **0** | **0** | **3** | **1** | **11** | **22** | **2** | **1** | **3** |
| ***Victorgorgia*** | **East** | **0** | **0** | **0** | **0** | **1** | **0** | **0** | **0** | **0** | **0** | **0** |
| ***Victorgorgia*** | **North** | **0** | **0** | **0** | **0** | **0** | **1** | **4** | **0** | **0** | **0** | **0** |
| ***Victorgorgia*** | **South** | **0** | **0** | **0** | **0** | **1** | **3** | **7** | **3** | **0** | **0** | **0** |

##### Supplemental Table S3.

Local Contribution to Beta Diversity for each dive. The LCBD represents the ecological comparative uniqueness of each sample. The p = value for each bin was assessed through permutation analysis (999 iterations) testing if the null hypothesis that genera distribution is random among the sampling depths. Blank cells were not covered by the dives.

| **Depth (m)** | **West** | **P value** | **East** | **P value** | **North** | **P value** | **South** | **P value** |
| --- | --- | --- | --- | --- | --- | --- | --- | --- |
| **240** | 0.01055821 | 0.054 | 0.01053052 | 0.057 | 0.00858532 | 0.298 | 0.01006325 | 0.121 |
| **270** | 0.00805435 | 0.448 | 0.01021841 | 0.091 | 0.00793561 | 0.468 | 0.00989966 | 0.119 |
| **300** | 0.0083673 | 0.342 | 0.00864487 | 0.295 | 0.00788643 | 0.464 | 0.0084734 | 0.339 |
| **330** | 0.0090314 | 0.212 | 0.00869924 | 0.263 | 0.0081948 | 0.409 | 0.00857671 | 0.303 |
| **360** | 0.00747817 | 0.605 | 0.0073957 | 0.651 | 0.00730431 | 0.668 | 0.00858532 | 0.298 |
| **390** | 0.00727077 | 0.699 | 0.00858532 | 0.31 | 0.00696166 | 0.767 | 0.00651281 | 0.854 |
| **420** | 0.00638528 | 0.868 | 0.00738118 | 0.645 | 0.00650819 | 0.872 | 0.00600253 | 0.926 |
| **450** | 0.00682924 | 0.804 | 0.00627381 | 0.897 | 0.00541583 | 0.966 | 0.00635738 | 0.887 |
| **480** | 0.00723586 | 0.717 | 0.00687236 | 0.78 | 0.00588666 | 0.939 | 0.00629876 | 0.881 |
| **510** | 0.00723586 | 0.695 | 0.00626187 | 0.886 | 0.00594171 | 0.95 | 0.00558561 | 0.961 |
| **540** | 0.00744296 | 0.659 | 0.00886612 | 0.249 | 0.00764536 | 0.572 | 0.00709199 | 0.729 |
| **570** | 0.00717039 | 0.717 | 0.00772243 | 0.565 | 0.00705541 | 0.748 | 0.00876228 | 0.3 |
| **600** | 0.01074697 | 0.028 | 0.00853901 | 0.301 | 0.00689112 | 0.783 | 0.00777145 | 0.529 |
| **750** | 0.00583402 | 0.95 | 0.00847706 | 0.326 | 0.01032445 | 0.088 | 0.01069218 | 0.066 |
| **780** | 0.00756151 | 0.595 | 0.00784163 | 0.529 | 0.00680404 | 0.8 | 0.01100839 | 0.025 |
| **810** | 0.00851202 | 0.33 | 0.00787259 | 0.483 | 0.00755021 | 0.631 | 0.0113664 | 0.009 |
| **840** | 0.00705522 | 0.765 | 0.00817198 | 0.403 | 0.00886612 | 0.256 | 0.01131041 | 0.009 |
| **870** | 0.00725551 | 0.702 | 0.00598095 | 0.938 | 0.0065599 | 0.848 | 0.00884393 | 0.265 |
| **900** | 0.00720663 | 0.723 | 0.00793272 | 0.466 | 0.01148559 | 0.002 | 0.00952776 | 0.167 |
| **930** | 0.00735553 | 0.678 | 0.00570298 | 0.965 | 0.00848703 | 0.314 | 0.00723586 | 0.689 |
| **960** | 0.00536527 | 0.96 | 0.00675389 | 0.813 | 0.00713557 | 0.718 | 0.00598204 | 0.935 |
| **990** | 0.00560929 | 0.958 | 0.00740992 | 0.651 | 0.00752544 | 0.635 | 0.00588986 | 0.941 |
| **1020** | 0.00689243 | 0.78 | 0.00806319 | 0.414 | 0.00796048 | 0.468 | 0.00587606 | 0.942 |
| **1050** | 0.00682304 | 0.814 | 0.01015673 | 0.1 | 0.01039125 | 0.068 | 0.00459338 | 0.98 |
| **1080** | 0.00765272 | 0.562 | 0.0066572 | 0.835 | 0.00723586 | 0.699 | 0.00544941 | 0.96 |
| **1110** | 0.00649042 | 0.88 | 0.00764892 | 0.58 | 0.00701761 | 0.77 | 0.00642882 | 0.882 |
| **1140** | 0.00688821 | 0.784 | 0.00894451 | 0.248 | 0.00723586 | 0.726 | 0.00912712 | 0.22 |
| **1170** | 0.0070776 | 0.732 | 0.01177894 | 0.001 | 0.00809427 | 0.429 | 0.00982934 | 0.134 |
| **1260** | 0.00978394 | 0.125 | 0.01057369 | 0.05 | 0.00723586 | 0.711 |  |  |
| **1290** | 0.00981308 | 0.142 |  |  | 0.0101406 | 0.119 |  |  |
| **1320** | 0.00781336 | 0.5 |  |  | 0.0106294 | 0.067 |  |  |
| **1380** | 0.00654144 | 0.866 |  |  | 0.01019403 | 0.09 |  |  |
| **1410** | 0.00764892 | 0.579 |  |  | 0.00968751 | 0.153 |  |  |
| **1440** | 0.00975518 | 0.154 |  |  |  |  |  |  |
| **1470** | 0.00963813 | 0.16 |  |  |  |  |  |  |
| **1500** | 0.00974644 | 0.161 |  |  |  |  |  |  |

Supplementary Table S4: Collections of deep-sea corals and sponges present at the target seamount. Collections with an MCZ Catalogue number have been deposited into the Invertebrate Zoology Collections at the Museum of Comparative Zoology at Harvard University (MCZIZ). Remaining collections are housed within the Rotjan Lab at Boston University (BU-RL). Missing latitude and longitude values were due to a intermittent technical problem on the ROV data logging system.

| **Repository** | **Cruise Sample Number** | **Dive Number** | **Seamount Flank** | **Date Collected (UTC)** | **Family** | **Species** | **Latitude (decimal degrees)** | **Longitude (decimal degrees)** | **Depth (m)** |
| --- | --- | --- | --- | --- | --- | --- | --- | --- | --- |
| MCZIZ169614 | FK21-476 | S0433 | West | 6/25/21 | Primnoidae | *Calyptrophora agassizii (coral)* | -1.61160047 | -175.2177 | 1152 |
| MCZIZ169615 | FK21-485 | S0433 | West | 6/26/21 | Primnoidae | *Narella alata (coral)* | -1.61225111 | -175.21005 | 746 |
| MCZIZ169616 | FK21-486 | S0433 | West | 6/26/21 | Primnoidae | *Narella studeri (coral)* | -1.61225308 | -175.21004 | 746 |
| MCZIZ169617 | FK21-487 | S0433 | West | 6/26/21 | Primnoidae | *Paracalyptrophora hawaiiensis (coral)* | -1.61256874 | -175.20853 | 590 |
| MCZIZ169618 | FK21-488 | S0433 | West | 6/26/21 | Stylasteridae | *Errina cheilopora (hydrozoan)* | -1.61228523 | -175.20783 | 513 |
| MCZIZ169619 | FK21-497 | S0433 | West | 6/26/21 | Dendrophyliidae | *Balanophyllia gemma (coral)* | -1.61151309 | -175.20414 | 242 |
| BU-RL | FK21-468 | S0433 | West | 6/25/21 | Chrysogorgiidae | *Metallogorgia melanotrichos (coral)* | -1.61363983 | -175.22358 | 1452 |
| BU-RL | FK21-469 | S0433 | West | 6/25/21 | Chrysogorgiidae | *Chrysogorgia* sp. *(coral)* | -1.61366493 | -175.22309 | 1431 |
| BU-RL | FK21-470 | S0433 | West | 6/25/21 | Chrysogorgiidae | *Chrysogorgia* sp. *(coral)* | -1.6136806 | -175.22292 | 1423 |
| BU-RL | FK21-471 | S0433 | West | 6/25/21 | Euplectellidae | *Walteria* sp. *(sponge)* | -1.61370269 | -175.22289 | 1420 |
| BU-RL | FK21-473 | S0433 | West | 6/25/21 | Paramuriceidae | Paramuriceidae sp. A *(coral)* | -1.61319479 | -175.22118 | 1345 |
| BU-RL | FK21-474 | S0433 | West | 6/25/21 | Plexauridae | *Swiftia* sp. *A (coral)* | -1.61292456 | -175.22077 | 1315 |
| BU-RL | FK21-479 | S0433 | West | 6/26/21 | Victorgorgiidae | *Trachythela* sp. *(coral)* | -1.61204366 | -175.2163 | 1075 |
| BU-RL | FK21-480 | S0433 | West | 6/26/21 | Paramuriceidae | *Paramuriceidae sp. B (coral)* | -1.61196093 | -175.21572 | 1047 |
| BU-RL | FK21-481 | S0433 | West | 6/26/21 | Bathyporidae | *Madrepora oculate (coral)* | -1.61193675 | -175.21531 | 1023 |
| BU-RL | FK21-482 | S0433 | West | 6/26/21 | Paramuriceidae | Paramuriceidae sp. C *(coral)* | -1.61172188 | -175.21423 | 965 |
| BU-RL | FK21-483 | S0433 | West | 6/26/21 | Chrysogorgiidae | *Metallogorgia* sp. 4 *(coral)* | -1.61175956 | -175.21404 | 953 |
| MCZIZ169621 | FK21-535 | S0434 | East | 6/27/21 | Aphrocallistidae | *Aphrocallistes Beatrix (sponge)* | -1.60984274 | -175.1998 | 249 |
| MCZIZ169620 | FK21-521 | S0434 | East | 6/26/21 | Caryophylliidae | *Vaughanella concinna (coral)* | -1.610362 | -175.18654 | 1213 |
| MCZIZ169622 | FK21-536 | S0434 | East | 6/27/21 | Agariciidae | *Dactylotrochus cervicornis (coral)* | -1.60990988 | -175.20096 | 219 |
| BU-RL | FK21-522 | S0434 | East | 6/26/21 | Caryophylliidae | *Vaughanella concinna (coral)* | -1.61040458 | -175.18696 | 1187 |
| BU-RL | FK21-523 | S0434 | East | 6/26/21 | Keratoisididae | Keratoisididae Clade C1 sp. *(coral)* | -1.60997605 | -175.19099 | 938 |
| BU-RL | FK21-525 | S0434 | East | 6/26/21 | Chrysogorgiidae | *Rhodaniridogorgia superba (coral)* | -1.61005799 | -175.19225 | 856 |
| BU-RL | FK21-526 | S0434 | East | 6/26/21 | Plexauridae | *Swiftia* sp. B *(coral)* | -1.60994592 | -175.19284 | 816 |
| BU-RL | FK21-527 | S0434 | East | 6/26/21 | Caryophylliidae | *Vaughanella* sp. *(coral)* | -1.61009445 | -175.19296 | 805 |
| BU-RL | FK21-529 | S0434 | East | 6/26/21 | Keratoisididae | Keratoisididae *(coral)* | -1.61000594 | -175.19323 | 783 |
| BU-RL | FK21-530 | S0434 | East | 6/26/21 | Primnoidae | *Calyptrophora* sp. *(coral)* | -1.6098696 | -175.19331 | 775 |
| BU-RL | FK21-531 | S0434 | East | 6/26/21 | Primnoidae | *Calyptrophora* sp. *(coral)* | -1.60987363 | -175.19331 | 775 |
| BU-RL | FK21-534 | S0434 | East | 6/26/21 | Paramuriceidae | *Acanthogorgia* sp. *(coral)* | -1.60946954 | -175.19697 | 454 |
| BU-RL | FK21-537 | S0434 | East | 6/27/21 | Paramuriceidae | *Imbricacis cf. squamata (coral)* | -1.60975033 | -175.20172 | 209 |
| MCZIZ169623 | FK21-560 | S0435 | North | 6/27/21 | Euplectellidae | Corbitellinae *(sponge)* | -1.59418157 | -175.20804 | 956 |
| MCZIZ169624 | FK21-561 | S0435 | North | 6/27/21 | Tretodictyidae | *Sclerothamanus* sp. *(sponge)* | -1.59545139 | -175.20811 | 852 |
| MCZIZ169625 | FK21-563 | S0435 | North | 6/27/21 | Primnoidae | *Narella alata (coral)* | -1.59577585 | -175.20781 | 825 |
| MCZIZ169626 | FK21-567 | S0435 | North | 6/27/21 | Primnoidae | *Paracalyptrophora hawaiiensis (coral)* | -1.59861543 | -175.20721 | 541 |
| MCZIZ169627 | FK21-572 | S0435 | North | 6/27/21 | Primnoidae | *Paracalyptrophora hawaiiensis (coral)* | -1.59862469 | -175.2072 | 541 |
| MCZIZ169628 | FK21-580 | S0435 | North | 6/27/21 | Dendrophyliidae | unidentifiable Dendrophyliidae *(coral)* | -1.60226412 | -175.20735 | 320 |
| MCZIZ169629 | FK21-581 | S0435 | North | 6/27/21 | Primnoidae | *Narella horrida (coral)* | -1.60312339 | -175.20718 | 297 |
| MCZIZ169630 | FK21-600 | S0435 | North | 6/27/21 | Dendrophyliidae | *Balanophyllia cornu (coral)* | -1.60312339 | -175.20718 | 297 |
| BU-RL | FK21-555 | S0435 | North | 6/27/21 | Coralliidae | *Hemicorallium* cf. *imperial (coral)* | -1.5888625 | -175.20869 | 1221 |
| BU-RL | FK21-556 | S0435 | North | 6/27/21 | Chrysogorgiidae | *Chrysogorgia* cf. *geniculata (coral)* | -1.58888744 | -175.2087 | 1219 |
| BU-RL | FK21-557 | S0435 | North | 6/27/21 | Coralliidae | Hemicorallium cf. *imperial (coral)* | -1.58912527 | -175.20873 | 1202 |
| BU-RL | FK21-562 | S0435 | North | 6/27/21 | Keratoisididae | Keratoisididae Clade C1 sp. *(coral)* | -1.59545139 | -175.20811 | 852 |
| BU-RL | FK21-565 | S0435 | North | 6/27/21 | Keratoisididae | Keratoisididae Clade C1 sp. *(coral)* |  |  | 815 |
| BU-RL | FK21-566 | S0435 | North | 6/27/21 | Chrysogorgiidae | *Rhodaniridogorgia* sp. *(coral)* |  |  | 806 |
| BU-RL | FK21-569 | S0435 | North | 6/28/21 | Dendrophylliidae | *Enallopsammia rostrata (coral)* | -1.59857801 | -175.2075 | 537 |
| BU-RL | FK21-579 | S0435 | North | 6/28/21 | Alcyoniidae | cf. *Eleutherobia* sp. *(coral)* | -1.60226479 | -175.20734 | 320 |
| BU-RL | FK21-582 | S0435 | North | 6/28/21 | Paramuriceidae | *Bebryce brunnea (coral)* | -1.60347811 | -175.20724 | 292 |
| BU-RL | FK21-584 | S0435 | North | 6/28/21 | Plexauridae | *Swiftia* sp. C *(coral)* | -1.60347572 | -175.20724 | 292 |
| BU-RL | FK21-585 | S0435 | North | 6/28/21 | Paramuriceidae | Paramuriceidae sp. D *(coral)* | -1.60365857 | -175.20691 | 285 |
| BU-RL | FK21-586 | S0435 | North | 6/28/21 | Paramuriceidae | Paramuriceidae sp. D *(coral)* | -1.60365928 | -175.20691 | 285 |
| MCZIZ169631 | FK21-607 | S0436 | South | 6/28/21 | Primnoidae | Calyptrophora sp. *(coral)* | -1.62676987 | -175.19727 | 996 |
| MCZIZ169632 | FK21-611 | S0436 | South | 6/28/21 | Stylasteridae | *Errina cheilopora (hydrozoan)* | -1.61914414 | -175.19924 | 456 |
| MCZIZ169633 | FK21-616 | S0436 | South | 6/28/21 | Micrabaciidae | *Letepsammia formosissima (coral)* | -1.61567575 | -175.2004 | 295 |
| BU-RL | FK21-605 | S0436 | South | 6/28/21 | Stylasteridae | *Lepidopora* sp. *(hydrozoan)* |  |  | 1023 |
| BU-RL | FK21-606 | S0436 | South | 6/28/21 | Victorgorgiidae | *Trachythela* sp. *(coral)* |  |  | 1006 |
| BU-RL | FK21-607 | S0436 | South | 6/28/21 | Primnoidae | *Calyptrophora* sp. *(coral)* |  |  | 996 |
| BU-RL | FK21-608 | S0436 | South | 6/28/21 | Paramuriceidae | *Acanthogorgia* sp. *(coral)* |  |  | 940 |
| BU-RL | FK21-614 | S0436 | South | 6/28/21 | Alcyoniidae | *Anthothela* sp. *(coral)* | -1.61793253 | -175.19974 | 381 |
| BU-RL | FK21-615 | S0436 | South | 6/28/21 | Micrabaciidae | *Letepsammia formosissima (coral)* | -1.61577558 | -175.20038 | 297 |
